# Multistage pH-responsive codelivery liposomal platform for synergistic cancer therapy

**DOI:** 10.1186/s12951-022-01383-z

**Published:** 2022-04-02

**Authors:** Ting Zhao, Ce Liang, Yanrong Zhao, Xiangdong Xue, Zhao Ma, Jinlong Qi, Haitao Shen, Shaokun Yang, Jia Zhang, Qingzhong Jia, Qing Du, Deying Cao, Bai Xiang, Hailin Zhang, Xianrong Qi

**Affiliations:** 1 Key Laboratory of Hebei Province for Innovative Drug Research and Evaluation, School of Pharmaceutical Sciences, Hebei Medical University, Shijiazhuang, 050017 Hebei China; 2 Department of Pharmacology, Hebei Medical University, Shijiazhuang, 050017 Hebei China; 3 Department of Hematology, Hebei Children’s Hospital, Hebei Medical University, Shijiazhuang, 050031 Hebei China; 4 School of Pharmaceutical Science, Shanghai Jiao Tong University, Shanghai, 200240 China; 5 Department of Medicinal Chemistry, Key Laboratory of Chemical Biology (MOE), School of Pharmaceutical Sciences, Cheeloo College of Medicine, Shandong University, Jinan, 250012 Shandong China; 6 Laboratory of Pathology, Hebei Medical University, Shijiazhuang, 050017 Hebei China; 7 School of Pharmaceutical Sciences, Peking University, Beijing, 100191 China

**Keywords:** Cancer therapy, pH-responsive, Liposome, Small interfering RNA, Docetaxel, Co-delivery system

## Abstract

**Background:**

Small interfering RNA (siRNA) is utilized as a potent agent for cancer therapy through regulating the expression of genes associated with tumors. While the widely application of siRNAs in cancer treatment is severely limited by their insufficient biological stability and its poor ability to penetrate cell membranes. Targeted delivery systems hold great promise to selectively deliver loaded drug to tumor site and reduce toxic side effect. However, the elevated tumor interstitial fluid pressure and efficient cytoplasmic release are still two significant obstacles to siRNA delivery. Co-delivery of chemotherapeutic drugs and siRNA represents a potential strategy which may achieve synergistic anticancer effect. Herein, we designed and synthesized a dual pH-responsive peptide (DPRP), which includes three units, a cell-penetrating domain (polyarginine), a polyanionic shielding domain (ehG)_n_, and an imine linkage between them. Based on the DPRP surface modification, we developed a pH-responsive liposomal system for co-delivering polo-like kinase-1 (PLK-1) specific siRNA and anticancer agent docetaxel (DTX), D-Lsi/DTX, to synergistically exhibit anti-tumor effect.

**Results:**

In contrast to the results at the physiological pH (7.4), D-Lsi/DTX lead to the enhanced penetration into tumor spheroid, the facilitated cellular uptake, the promoted escape from endosomes/lysosomes, the improved distribution into cytoplasm, and the increased cellular apoptosis under mildly acidic condition (pH 6.5). Moreover, both in vitro and in vivo study indicated that D-Lsi/DTX had a therapeutic advantage over other control liposomes. We provided clear evidence that liposomal system co-delivering si*PLK-1* and DTX could significantly downregulate expression of PLK-1 and inhibit tumor growth without detectable toxic side effect, compared with si*PLK-1*-loaded liposomes, DTX-loaded liposomes, and the combinatorial administration.

**Conclusion:**

These results demonstrate great potential of the combined chemo/gene therapy based on the multistage pH-responsive codelivery liposomal platform for synergistic tumor treatment.

**Graphical Abstract:**

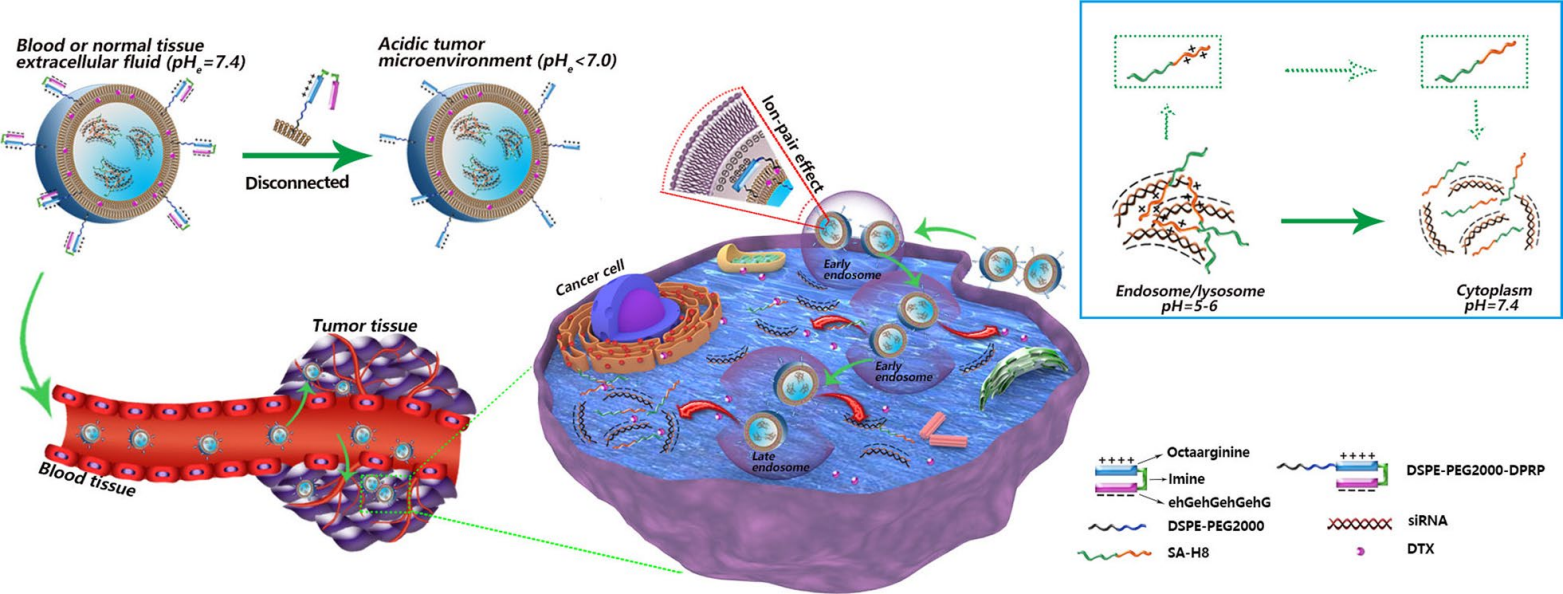

**Supplementary Information:**

The online version contains supplementary material available at 10.1186/s12951-022-01383-z.

## Background

Malignant tumors are the second leading cause of human death worldwide. Generally, the clinical treatment of advanced cancer mainly relies on traditional therapy (*e.g.,* chemotherapies). However, chemotherapy outcomes remain discouraging because of the poor specificity and the high systemic toxicity of chemotherapeutic drugs [1]. As an approach to explore more specific tumor treatment methods, many researchers are committed to the application of RNA interference (RNAi) [2]. Typically, small interfering RNA (siRNA)-based therapeutics represent an important tool to modulate the expression of genes related to tumor initiation, progression and metastasis [3].

However, the clinical application of siRNAs remains extremely challenging due to their low biological stability, unfavorable pharmacokinetics, and limited cellular uptake [4]. Cationic liposomes, one of the most widely validated carriers currently in use, have been developed for siRNA delivery to overcome these defects [5]. The further introduction of poly (ethylene glycol) (PEG) onto the liposome surface, avoids the recognition and clearance of nanocarriers by the reticuloendothelial system, which endows the delivery system with extended circulation half-lives and improved access to tumor regions through the enhanced permeability and retention (EPR) effect [6, 7]. Unfortunately, PEGylation has been reported to reduce targeting specificity [8], and inhibit both efficient cellular uptake and subsequent endosomal escape [9]. Furthermore, for effective siRNA condensation, most cationic liposomal systems have excessive positive charges, which might hamper cytosolic siRNA release due to electrostatic interactions [4]. In addition, solid tumors have increased interstitial fluid pressure (IFP) due to a dense extracellular matrix, rapid proliferation of cancer cells, increased intratumor vascular permeability, and poor lymphatic drainage around the blood vessels [10]. The elevated IFP results in blood stasis and reversal of blood flow, which hinders the intratumor penetration of nanocarriers. Thus, the development of a smart and efficient delivery platform for siRNA delivery with increased specificity for the tumor, deep tissue penetration, increased cellular uptake, and beneficial intracellular trafficking, including endosomal escape and cytoplasmic release, is urgently needed.

Cell-penetrating peptides (CPPs), also called “Trojan Horse” peptides, are a class of positively-charged peptides, typically with a size of 5–30 amino acids, such as human immunodeficiency virus (HIV)-1 transactivator of transcription (Tat)-derived peptide and artificial oligoarginine peptides [4]. Due to their interaction with the negatively-charged plasma membrane, CPPs facilitate the cellular internalization of a variety of cargoes (*e.g.,* DNA, siRNA, nanoparticles) [11, 12], thereby dramatically increasing their in vitro delivery efficiency. However, the lack of specificity for particular cells or tissues, severely hinders the extensive application of CPPs in the systemic delivery of cargoes in vivo [13]. To tame the wildness of the “Trojan Horse” peptide, several microenvironment-responsive strategies have been developed to achieve ‘off–on’ switches to that increase CPP potency in response to endogenous triggers (*e.g.,* pH-change [14, 15] and enzymatic activity [16, 17]). Through the charge-guided masking of oligoarginine, r8/r9, our research team have constructed a series of protease-cleavable activatable CPPs, that achieved the tumor selective delivery of diverse cargoes, including nanocarriers and chemotherapeutic molecules [17–22]. Notably, the tamed “Trojan Horse” might endow the delivered agents with both enhanced tumor penetration and increased endosomal/lysosomal escape following the protease-triggered demasking of oligoarginine.

The physiological pH of normal tissues and blood is approximately 7.4, while the extracellular pH of tumor tissue is weakly acidic (pH 6.5–6.8) [23], due to increased glucose uptake and metabolism, a phenomenon known as the Warburg effect [24]. Accordingly, the pH difference between normal and tumor tissues has been widely utilized as a triggering signal for the design of delivery systems based on extracellular acid-labile bonds, such as imine, amide, acetal and borate ester bonds [25–27]. Among these functionalities, the acid-degradable imine functionality has shown promising results for the pH-responsive delivery of anticancer agents [28–30]. For instance, a pH-responsive PEG-lipid derivative was constructed through imine-forming reaction to exploit the low extracellular pH (~ 6.5) in the tumor microenvironment. Following surface modification with this imine-containing amphiphilic molecule, liposomes and solid lipid nanoparticles were developed to encapsulate irinotecan and microRNA-200, respectively. These specially-designed nanocarriers displayed pH-responsive release, cellular uptake, and intracellular distribution in acidic pH of colon cancer HCT116 cells. Moreover, in vivo studies using tumor-bearing mice, indicated that dual-delivery of irinotecan and microRNA-200 achieved positive therapeutic outcomes by suppressing colorectal tumor growth and reducing systemic toxicity [31].

**Scheme 1:**
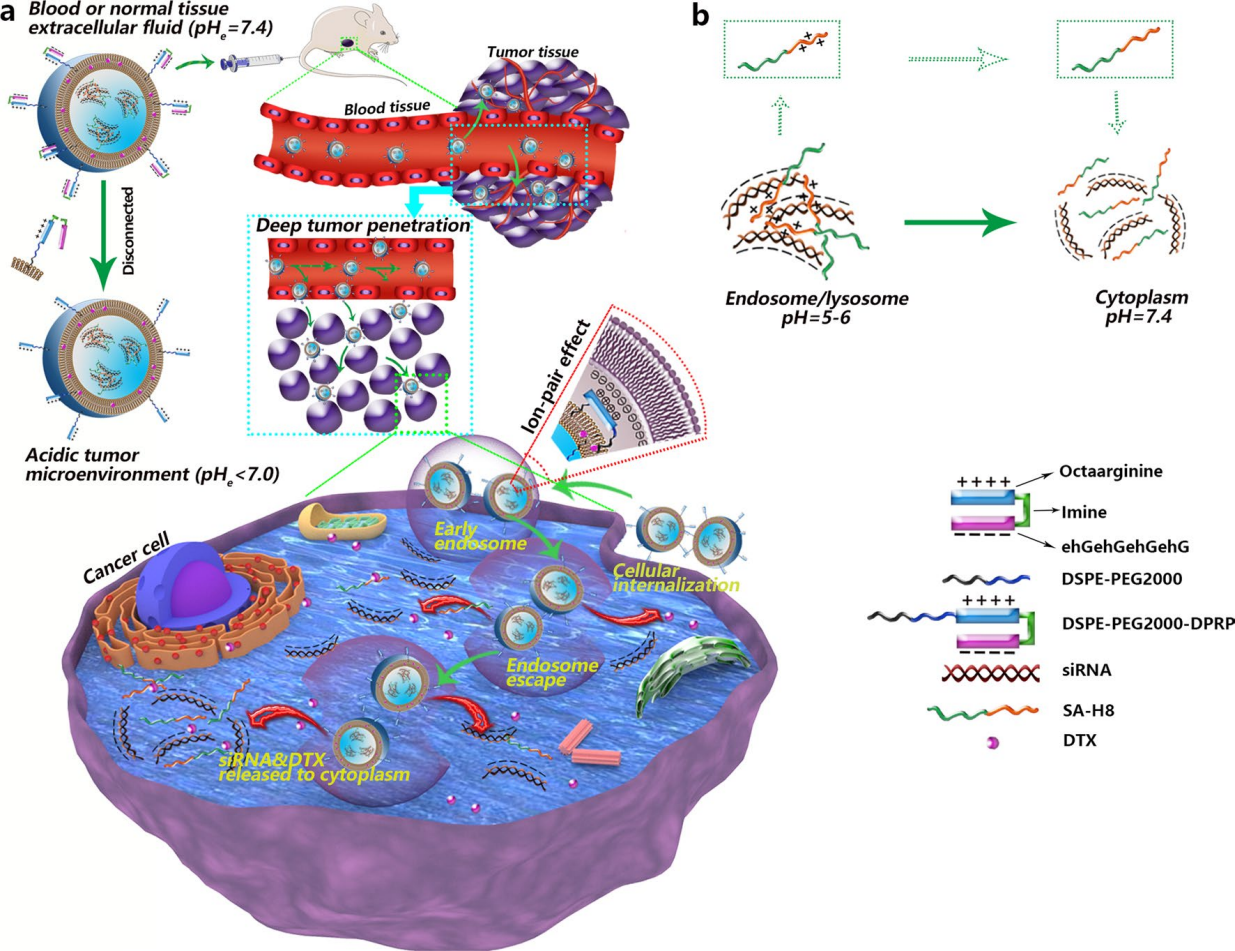
Multistage pH-responsive co-delivery liposome and its tumor-targeted delivery strategy. **a** The acidic pH_e_ in the tumor microenvironment splits DPRP at the imine site and detaches the shielding domain from the CPP section, thereby recovering the potency of CPP for enhanced cellular internalization and endosome/lysosome escape. **b** In the acidic environment of endosomes/lysosomes (pH 5.0–6.0), the anionic siRNA forms a complex with the cationic SA-H8 by electrostatic interactions. When the complexed siRNA delivered to cytoplasm (pH 7.4), histidines in SA-H8 undergo transformation into their uncharged form, resulting in the dissociation of the complex and efficient release of siRNA

Here, by taking full advantage of microenvironmental pH changes that occur during nanocarrier-mediated systemic delivery of siRNA for cancer treatment, we developed a novel multistage pH-responsive liposomal system for the codelivery of polo-like kinase-1 specific siRNA (si*PLK-1*) and docetaxel (DTX) (Scheme 1), which was mainly based on a dual pH-responsive peptide (DPRP). DPRP is comprised of three segments, a cell-penetrating domain (octaarginine), a polyanionic shielding domain (ehGehGehGehG), and an acidic pH-cleavable imine linker, which was used to connect the other two parts. The basic rationale is described below, in the blood circulation at pH 7.4, both glutamic acid and histidine residues present in the shielding domain remain negatively charged due to their lower pKa values [32], and then the surface polycationic moieties in octaarginine experience charge-shielding, because the penetration efficacy of the cell-penetrating domain is substantially diminished. Once approaching the tumor area with a reduced pH, the DPRP would be subject to breakage due to acid-catalyzed hydrolysis of the imine bonds. Meanwhile, protonation of the imidazole group results in a net positive charge of histidine in this mildly acidic environment [14], which substantially decreases the gross negative charges of the shielding domain, leading to its subsequent detachment from polyarginine fractions in the DPRP. Correspondingly, the cell-penetrating domain regains sufficient positive charges, and is thus conducive to deep tumor penetration and enhanced cellular entry of the codelivery system, facilitating the subsequent escape of the loaded siRNAs from endosomal/lysosomal compartments. Additionally, stearylated-octahistidine (SA-H8) was employed to compress siRNAs and achieve cytoplasmic release. In the acidic environment of endosomes/lysosomes (pH 5.0–6.0), the anionic siRNA forms a complex with cationic SA-H8 through electrostatic interactions. When the complexed siRNA was delivered to the cytoplasm (pH 7.4), histidines in SA-H8 transformed into their uncharged form due to the deprotonation of imidazole groups, resulting in the dissociation of the complex and efficient release of the siRNA. Thus, the multistage pH-responsive codelivery platform may endow the loaded therapeutic agents, siRNA and DTX, with increased tumor selectivity and enhanced tumor penetration and cellular uptake, thereby achieving effective endosomal escape and efficient cytoplasmic release of the encapsulated siRNA.

## Results and discussion

### Synthesis and identification of DPRP

**Fig. 1:**
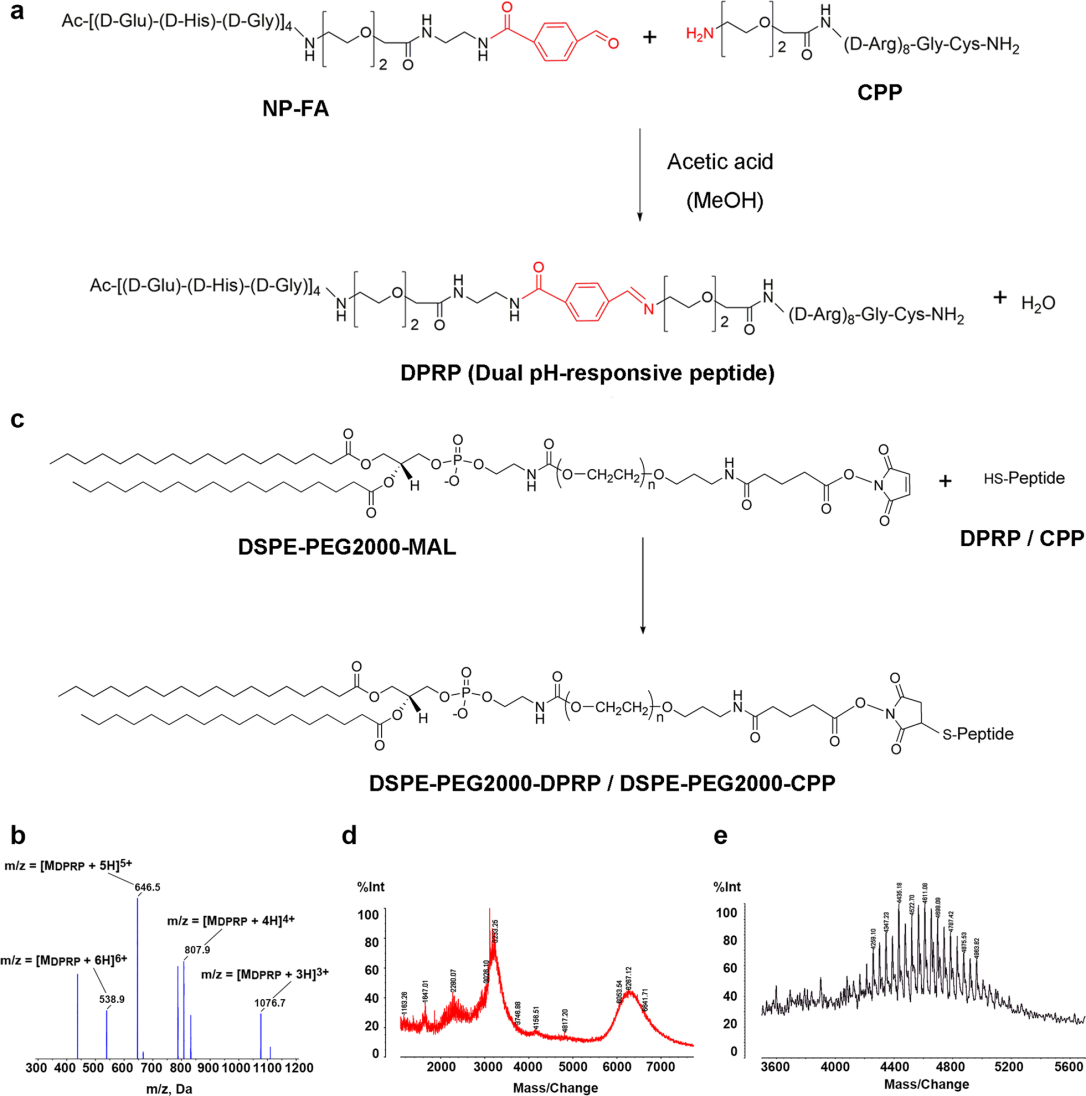
Characterization of DPRP and DSPE-PEG2000-DPRP/DSPE-PEG2000-CPP. **a** Principle of DPRP synthesis. **b** The positive ion electrospray ionization mass spectrum of DPRP. **c** Principle of the synthesis of DSPE-PEG2000-DPRP/DSPE-PEG2000-CPP. **d** MALDI-TOF mass spectra of DSPE-PEG2000-DPRP and (**e**) DSPE-PEG2000-CPP

The DPRP sequence (Ac-CGehGehGehGehGG-benzoic imine-rrrrrrrrGC) includes three units: polyarginine (octaarginine), the pH-responsive imine bond, and the polyanionic shielding domain (ehG)_n._ Polyarginine is a well-known CPP, that facilitates the entry of molecules into cells without requiring specific receptors [33]. The polyarginine peptide is temporarily inert due to the attenuated CPP through the electrostatic interaction of the polyanionic shielding peptides, and its activation is triggered in an acidic tumor microenvironment. DPRP was synthesized using the steps shown in Fig. 1a. Briefly, imine bond-containing DPRP was prepared via a Schiff reaction between the carbonyl group of negatively-charged peptide-4-formylbenzoic acid (NP-FA) and the primary amine group of CPP in the presence of acetic acid. The structure of DPRP was determined based on mass spectra (Fig. 1b). The [M + 3H]^3+^ (m/z 1076.7), [M + 4H]^4+^ (m/z 807.9), [M + 5H]^5+^ (m/z 646.4) and [M + 6H]^6+^ (m/z 538.7) peaks were consistent with the calculated values, suggesting that DPRP was successfully synthesized.

### Acid sensitivity of DPRP

The stability of DPRP was investigated at varying pH values prior to the conjugation of peptide to PEGylated lipids. According to the design strategy, cleavage of the acid-labile peptide sequence could occur at the imine bond and form two sections (octaarginine and (ehG)_n_) in the acidic tumor microenvironment. Consequently, two newly formed peaks that would be differentially retained from DPRP were detected in the HPLC chromatograph during the treatment of the DPRP sample at pH 6.5. One of them eluted at the same retention time (~ 7.2 min) as the polyarginine-containing peptide, indicating that the cleavage of DPRP was triggered at the predicted site. As shown in Additional file 1: Figure S1, the time required for splitting half of the peptide was 4.6 h, and the peak area of DPRP was decreased to ~ 5% of its original level following an incubation for 24 h. However, DPRP exhibited higher stability with a half-life greater than 30 h at physiological pH (pH 7.4) (Additional file 1: Figure S1). Based on these results, the imine bond exhibited a pH response in the tumor cell environment and maintained high stability under physiological conditions. The difference in stability at different pH values is likely ascribed to the pH response of the imine-based DPRP.

### Synthesis and identification of functional conjugates

The synthesis of DSPE-PEG2000-DPRP/DSPE-PEG2000-CPP was performed using a Michael addition reaction between the maleimide groups of DSPE-PEG2000-MAL and the thiol groups of DPRP/CPP (Fig. 1c). The two peaks centered at m/z 6267.12 (Fig. 1d) and 4611.08 (Fig. 1e) are consistent with the calculated mean MW of DSPE-PEG2000-DPRP and DSPE-PEG2000-CPP, respectively, which verified the successful preparation of the two functional conjugates.

### Preparation and characterization of liposomal formulations

**Fig. 2:**
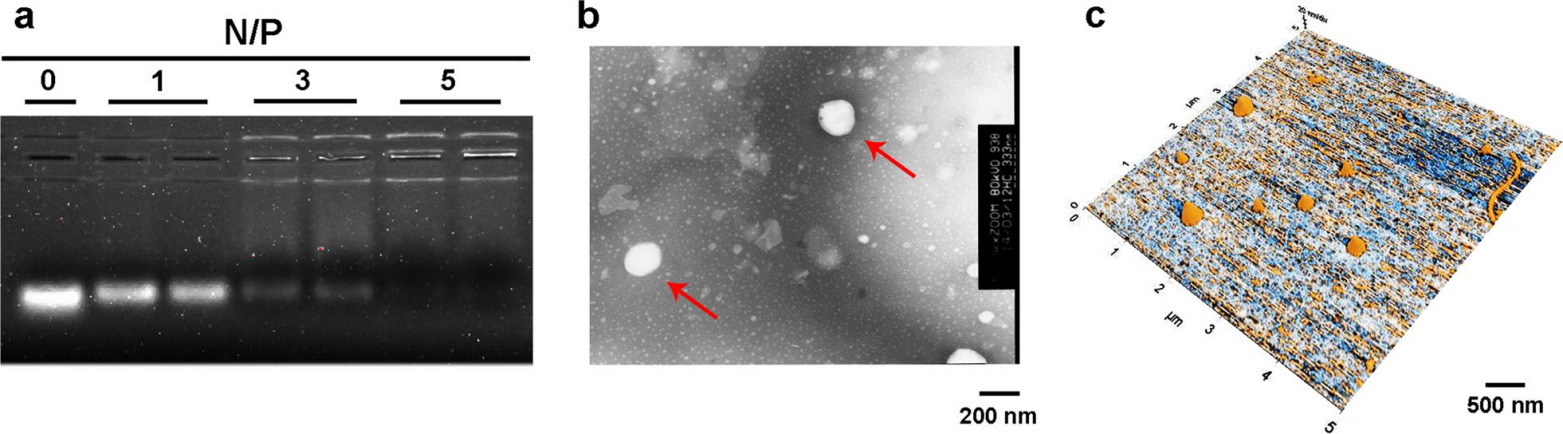
Characterization of liposomes. **a** Binding of SA-R8 to siRNA at different N/P ratios, as evidenced by the AGE assay. **b** TEM image of D-L liposomes. **c** AFM topographic image of D-L liposomes

According to our design principles, complexes were formed by the electrostatic interaction between positively charged SA-R8 (or SA-H8) and negatively charged siRNA. As indicated in Additional file 1: Figure S2a, we performed carbodiimide-mediated coupling between R8/H8 and SA and identified the structures of SA-R8 and SA-H8 using mass spectrometry (Additional file 1: Figure S2b and S2c). In an attempt to develop the optimal complexes, the best molar ratio of nitrogen in SA-R8/phosphate in siRNA (N/P ratio) was explored using an agarose gel electrophoresis (AGE) assay. The siRNA was entirely compressed into the complex at an N/P ratio of 5:1 (Fig. 2a).

DTX-free and DTX-loaded liposomes were prepared via the thin-film hydration method. The complex SA-R8/siRNA and SA-H8/siRNA was individually incubated with liposomes to obtain siRNA-loaded or siRNA/DTX-loaded liposomal formulations. Afterward, the liposomes underwent various modifications with PEG, PEG/CPP and PEG/DPRP via the postinsertion method, thus forming three types of liposomes: normal liposomes (N-L), CPP-modified liposomes (C-L) and DPRP-modified liposomes (D-L). Drug encapsulation efficiency (EE) of DTX was 95.9 ± 0.52%, and drug loading content (DLC) was 4.83 ± 0.026%.

The particle size of the liposomes was characterized using dynamic light scattering (DLS), as shown in Table 1. Size and zeta potential are two key parameters that determine the distribution of nanocarriers in the body. All types of liposomes had an average diameter of approximately 180–200 nm and a polydispersity index (PDI) ranging from 0.090 to 0.220. The zeta potential represents the electrostatic charge on the surface of the particles. As a result, the zeta potential of D-L (~ − 36 mV) was less than that of C-L (~ − 28 mV) but approximately the same as N-L (~ − 38 mV), which supported the hypothesis that the positively charged polycationic cell-penetrating domain experienced effective neutralization by the polyanionic shielding domain. The transmission electron microscopy (TEM) (Fig. 2b) and atomic force microscopy (AFM) images (Fig. 2c) showed that the liposomes were approximately spherical in shape with a uniform size distribution, consistent with the data obtained from the DLS analysis.

**Table 1:** Physicochemical properties of liposomes carrying siRNA or siRNA/DTX (n = 3)

	Functional arms [mol ratio of total lipid]	Diameter [nm]	PDI	Zeta-potential [mV]
N-L_R_/si	–	184.5 ± 2.3	0.098 ± 0.012	− 38.8 ± 0.78
C-L_R_/si	DSPE-PEG2000-CPP [5%]	191.6 ± 3.7	0.133 ± 0.016	− 28.4 ± 1.24
D-L_R_/si	DSPE-PEG2000-DPRP [5%]	202.9 ± 1.7	0.187 ± 0.023	− 34.3 ± 1.21
D-L_H_/si	DSPE-PEG2000-DPRP [5%]	209.9 ± 0.6	0.194 ± 0.021	− 36.4 ± 2.20
D-L_H_/si-DTX	DSPE-PEG2000-DPRP [5%]	211.3 ± 1.6	0.219 ± 0.012	− 36.1 ± 1.46

The data are expressed as the mean ± SD value for at least three different preparations

### Cellular internalization

**Fig. 3:**
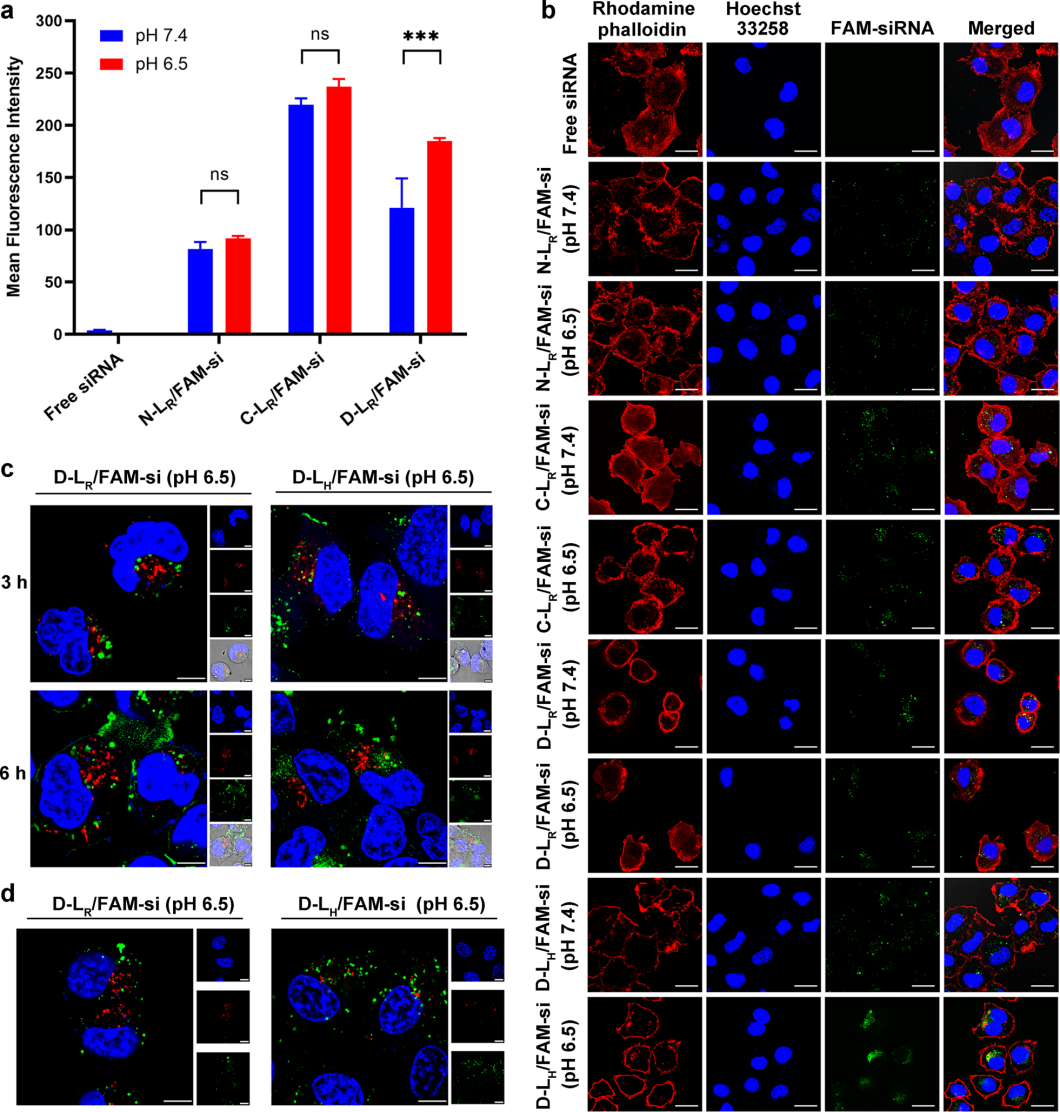
Cell uptake and intracellular trafficking of liposomal FAM-siRNA. **a** Flow cytometry measurement of liposomal FAM-siRNA uptake by MCF-7 cells. The FAM-siRNA concentration was 100 nM (n = 3). ***P < 0.001. **b** Confocal laser scanning microscopy (CLSM) analysis of liposomal FAM-siRNA uptake by MCF-7 cells. The FAM-siRNA concentration was 200 nM. Scale bar, 50 µm. **c** Intracellular trafficking of FAM-siRNA and the colocalization of FAM-siRNA with early endosomes in MCF-7 cells. The FAM-siRNA concentration was 200 nM. Scale bar, 7.5 µm. **d** Intracellular trafficking of FAM-siRNA and the colocalization of FAM-siRNA with late endosomes/lysosomes in MCF-7 cells. The FAM-siRNA concentration was 200 nM. Scale bar, 10 µm. Cell nuclei, F-actin, early endosomes and late endosomes/lysosomes were counterstained with Hoechst 33,258 (blue), rhodamine-phalloidin (red), CellLight Early Endosomes-RFP BacMam 2.0 (red) and LysoTracker Red (red), respectively. FAM-siRNA fluorescence (green) was recorded

MCF-7 cells were incubated with either free or formulated FAM-siRNA (N-L_R_/FAM-si, C-L_R_/FAM-si and D-L_R_/FAM-si) at pH 6.5 and 7.4 to systematically investigate the potential effects of various liposome formulations on the cellular uptake of siRNA. As presented in Fig. 3a, no obvious fluorescence signal was detected in the free siRNA group, indicating that free siRNA rarely penetrates cell membranes due to the large molecular weight and high density of negative charges [5]. Among all liposomal formulations, C-L_R_/FAM-si resulted in the greatest uptake of FAM-siRNA (Fig. 3a), indicating the increased cell membrane penetration of liposomes mediated by CPP. However, compared with the result obtained for C-L_R_/FAM-si at pH 7.4, the mean fluorescence intensity detected for D-L_R_/FAM-si was significantly reduced (*P* < 0.001), which inferred that the positively charged CPP section underwent substantial neutralization by the shielding domain. In contrast to the data collected at pH 7.4, only D-L_R_/FAM-si resulted in significantly enhanced cellular uptake of siRNA at pH 6.5 (*P* < 0.001), supporting the expected activation of CPP under acidic conditions.

The uptake of liposomal siRNA was also detected using confocal laser-scanning microscopy (CLSM) (Fig. 3b). Among all experimental groups, only D-L_R_/FAM-si presented significantly stronger green fluorescence at pH 6.5 than at pH 7.4, indicating the recovery of CPP under acidic conditions. These results were consistent with the flow cytometry data described above. In addition, compared with the image of cells treated with D-L_R_/FAM-si, the FAM-siRNA loaded in D-L_H_/FAM-si was dispersed in the cytoplasm to a much greater extent, which might be attributed to the transition of the histidine-containing domain to an electrically neutral state.

### Intracellular trafficking of D-L containing siRNA

MCF-7 cells were exposed to D-L at pH 6.5 for 3 h or 6 h to investigate the intracellular transport of the siRNA. As shown in Fig. 3c, intracellular FAM-siRNA (green) partially colocalized with early endosomes (red), indicating that liposomal FAM-siRNA entered MCF-7 cells through an endocytosis-included mechanism. In obvious contrast to the images collected after 3 h of incubation, a portion of the scattered FAM-siRNA signal was observed in the cytoplasm following 6 h of incubation, which suggested the occurrence of endosomal/lysosomal escape. Compared with D-L_R_/FAM-si, the siRNA formulated in D-L_H_/FAM-si was more dispersed, which indicated the detachment of siRNA from complexes and the subsequent diffusion into cytoplasm.

Based on the labeling of late endosomes/lysosomes with LysoTracker Red, partial overlap between FAM-siRNA and late endosomes/lysosomes was observed after 3 h of incubation at pH 6.5 (Fig. 3d). However, the colocalization of the siRNA with late endosomes/lysosomes was rarely detected following 6 h of treatment, and most of the internalized siRNA diffused into the cytoplasm (Fig. 3d). Based on these results, both D-L_R_/FAM-si and D-L_H_/FAM-si achieved endosomal escape of FAM-siRNA over time, which might be attributed to the ion-pair effect exerted by the recovered polyarginine domain. Similar results were observed for the intracellular translocation of other polyarginine-mediated delivery systems, as described in our previous publications [15, 17]. In addition to successful escape from acidic organelles, namely, endosomes and lysosomes, the efficient release of siRNAs in the cytoplasm is a prerequisite for subsequent gene silencing [5]. Apparently, the D-L_H_/FAM-si resulted in the greatest amounts of internalized siRNAs with a much higher dispersion compared to D-L_R_/FAM-si, indicating that SA-H8 might be preferable to SA-R8 for siRNA delivery.

**Fig. 4:**
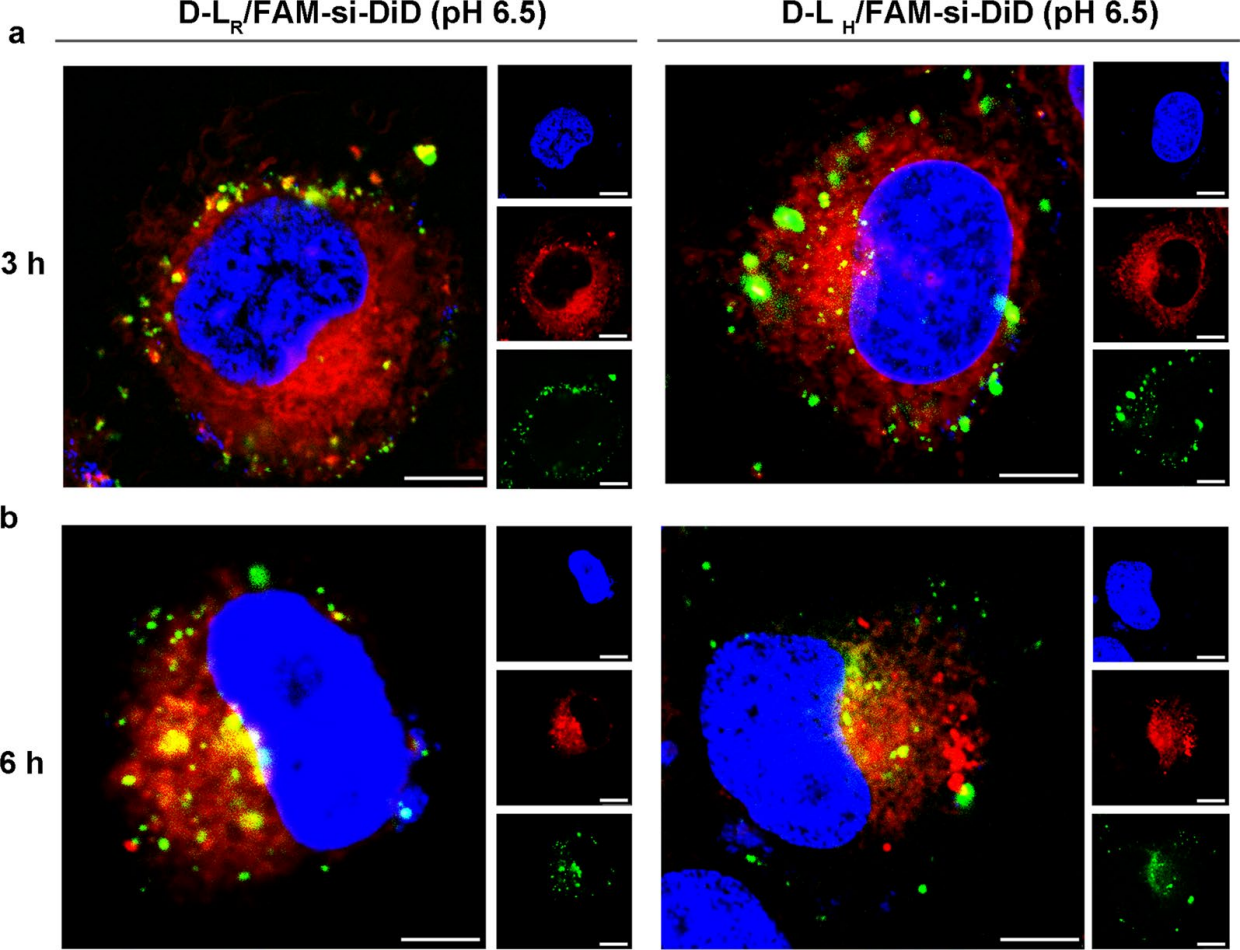
Intracellular uptake and distribution of FAM-siRNA and DiD in MCF-7 cells after an incubation with double-labeled liposomes for (**a**) 3 h and (**b**) 6 h. The FAM-siRNA and DiD concentrations were 400 nM and 680 ng mL^−1^, respectively. Scale bar, 7.5 µm. Cell nuclei and early endosomes were counterstained with Hoechst 33,258 (blue) and CellLight Early Endosomes-RFP BacMam 2.0 (red), respectively. FAM-siRNA fluorescence (green) and DiD fluorescence (red) were recorded

DTX prevents cell division and induces apoptosis by increasing the polymerization of tubulin and inhibiting the depolymerization of microtubules in the cytoplasm [34]. Notably, siRNAs escape into the cytoplasm to induce a specific gene silencing effect [5]. Accordingly, nanocarriers coloaded with DTX and siRNA exhibited the synergistic function of inducing cell apoptosis and gene suppression only when these two payloads were exclusively located in the cytoplasm. DiD, a lipophilic fluorescent probe, was utilized in the formulation instead to investigate the intracellular trafficking of DTX. As shown in Fig. 4a, both DiD and FAM-siRNA were effectively translocated into cells. DiD exhibited much faster diffusion and a more uniform distribution throughout the entire cytoplasm at 3 h; however, most of the FAM-siRNA was located in the cytoplasm in a highly aggregated form, suggesting the collapse of the liposomal membrane and the release of the complexes. Thus, the codelivery system D-L_R_/si-DTX provides the simultaneous encapsulation and delivery of gene therapy agents (siRNA) and chemotherapy drugs (DTX) to tumor cells. For D-L_H_/FAM-si, the green spots scattered gradually and were more widely distributed than those of D-L_R_/FAM-si after a further 3 h treatment (Fig. 4b), which confirmed the release of the complexed siRNA and the strength of SA-H8.

### Gene silencing analysis

**Fig. 5:**
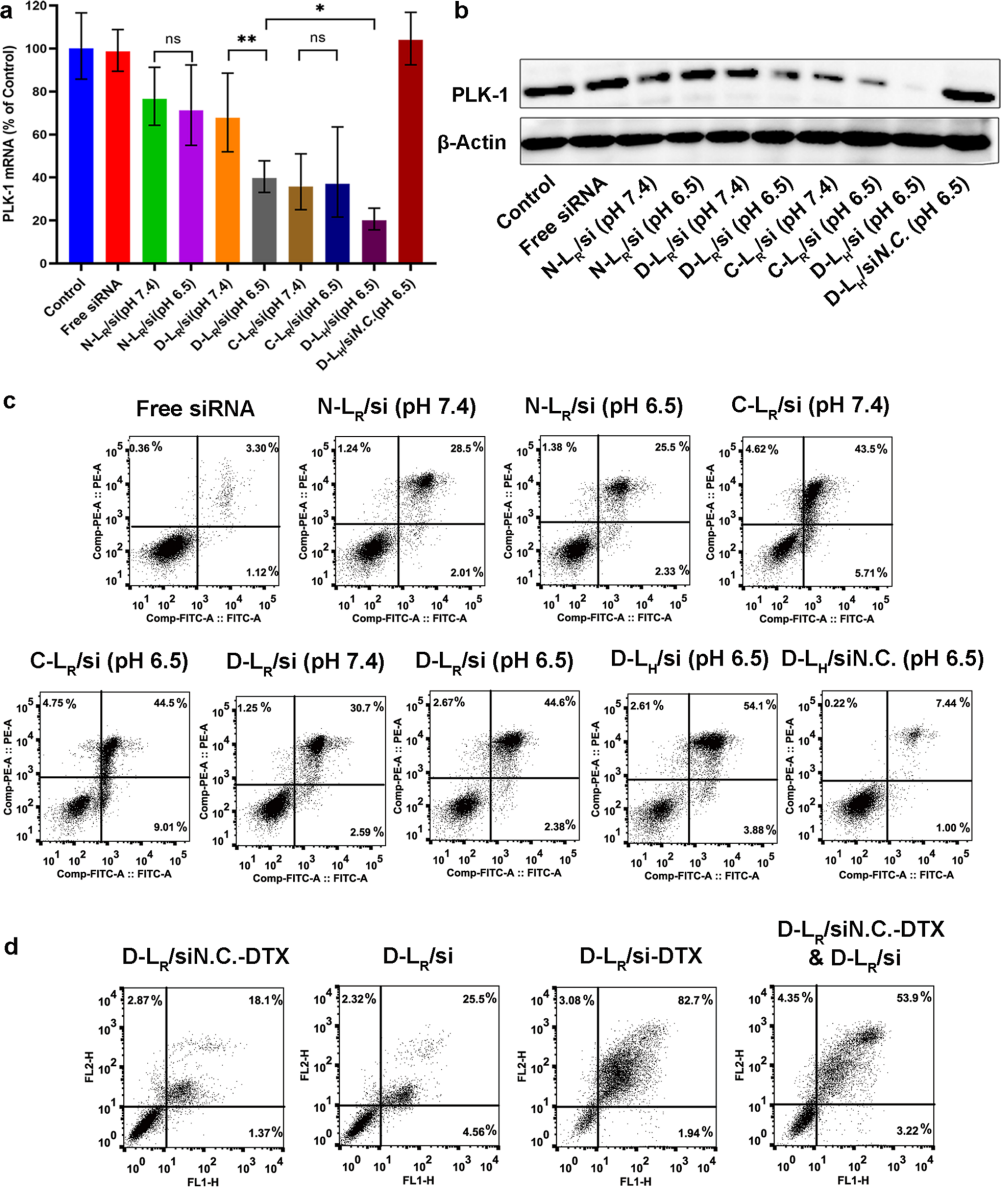
Gene silencing efficacy and cytotoxicity evaluation. **a** Expression of the PLK-1 mRNA determined using qRT–PCR. **b** Expression of the PLK-1 protein determined using western blot analysis. Cell apoptosis following exposure to (**c**) liposomes carrying individual drugs and (**d**) codelivery liposomes. Early apoptotic cells are shown in the lower right quadrant, and late apoptotic cells are shown in the upper right quadrant. *P < 0.05 and **P < 0.01

As one of the most important regulators of mitosis in mammalian cells, PLK-1 is overexpressed in various human tumors, indicating its involvement in carcinogenesis and its potential as a target for treating tumors [35]. We conducted qRT–PCR in MCF-7 cells to assess the downregulation of the target gene PLK-1 by siRNA-loaded liposomes. As shown in Fig. 5a, compared with C-L_R_/si, D-L_R_/si resulted in significantly different levels of PLK-1 mRNA in response to the change in pH, with the expression observed at pH 6.5 being much lower than that at pH 7.4. This difference might be attributed to the improved internalization of the formulated si*PLK-1* at acidic pH (Fig. 3a, b) and its subsequent effective escape from the endosome/lysosome (Fig. 3c, d) caused by the acidic hydrolysis of the pH-responsive imine bond. Further investigations revealed that the transfection of MCF-7 cells with D-L_H_/si resulted in greater downregulation of the target mRNA compared with D-L_R_/si treatment (Fig. 5a), suggesting the improved cytoplasmic release of si*PLK-1*, as confirmed in Fig. 4b.

Next, we investigated whether this reduction in the PLK-1 mRNA level was accompanied by a decrease in PLK-1 protein levels in MCF-7 cells by performing western blot analyses. As shown in Fig. 5b, the delivery of si*PLK-1* by liposomes exclusively reduced PLK-1 protein expression. However, treatment with both free si*PLK-1* and si*N.C.*-loaded D-L exerted a negligible effect on reducing PLK-1 protein expression, supporting the hypothesis that no nonspecific gene silencing occurred and that naked siRNAs did not passively translocate across the cell membrane due to their high molecular weight and negative charge [36, 37]. The expression of the PLK-1 protein was markedly inhibited only in MCF-7 cells incubated with si*PLK-1*-loaded D-L at pH 6.5 but not at pH 7.4. Additionally, the lowest PLK-1 protein expression was detected in cells treated with D-L_H_/si, providing further evidence for the anticipated pH responsivity of D-L and SA-H8 to the extracellular and intracellular tumor microenvironments, respectively.

### Cell apoptosis assay

PLK-1 knockdown has been shown to induce cell apoptosis in a variety of tumors [35]. Therefore, we evaluated the apoptotic activity after treating MCF-7 cells with formulated si*PLK-1* at a final concentration of 100 nM. As illustrated in Fig. 5c, C-L_R_/si induced the highest level of apoptosis (~ 49%), while D-L_R_/si resulted in significantly reduced apoptosis (~ 33%) at pH 7.4, which could be attributed to the reduced cellular uptake of liposomal si*PLK-1* due to electrostatic masking of the shielding domain, as observed in Fig. 3a and b. Further investigations were conducted to evaluate whether the induction of cell apoptosis by the formulated si*PLK-1* exhibited a pH response. When the medium pH was adjusted from 7.4 to 6.5, no obvious difference in the apoptotic rate was observed in either N-L_R_/si- or C-L_R_/si-treated cells. However, more cells exposed to D-L_R_/si underwent apoptosis at pH 6.5 than at pH 7.4, reflecting activation of the shielded CPP. Additionally, the induction of cell apoptosis by D-L_H_/si was markedly increased (~ 58% of apoptotic cells) compared to D-L_R_/si (~ 47% of apoptotic cells), potentially due to the increased release of si*PLK-1* in cytoplasm, as presented in Fig. 3d. These data supported the pH responsivity of both DPRP and SA-H8, which obviously facilitated RNAi-mediated gene silencing. Moreover, the correlation between apoptosis and gene silencing confirmed that the reduction in PLK-1 mRNA expression is responsible for the downregulated PLK-1 protein expression in MCF-7 cells and the induction of cell apoptosis.

MCF-7 cells were incubated with formulations coencapsulating si*PLK-1* and DTX to investigate their synergistic proapoptotic effects. As indicated in Fig. 5d, transfection with D-L_R_/si-DTX resulted in an increased percentage (~ 84%) of apoptotic cells that was much higher than that of the combinatorial delivery system (D-L_R_/si*N.C.*-DTX & D-L_R_/si, ∼57% of apoptotic cells), which correlated well with the result of cell proliferation assay (Additional file 1: Figure S3) and revealed a synergistic effect on cancer cells due to simultaneous delivery of si*PLK-1* and DTX. As a microtubule-stabilizing agent, DTX preferentially induces G2/M arrest of the cell cycle and subsequently causes apoptosis [38]. Moreover, PLK-1 knockdown leads to tumor growth arrest and increases the sensitivity of MCF-7 cells to taxane treatment [39]. Thus, codelivery of si*PLK-1* and DTX arrests the cell cycle, which subsequently achieves increased apoptosis [40].

### Penetration and inhibition on three-dimensional tumor spheroids

**Fig. 6:**
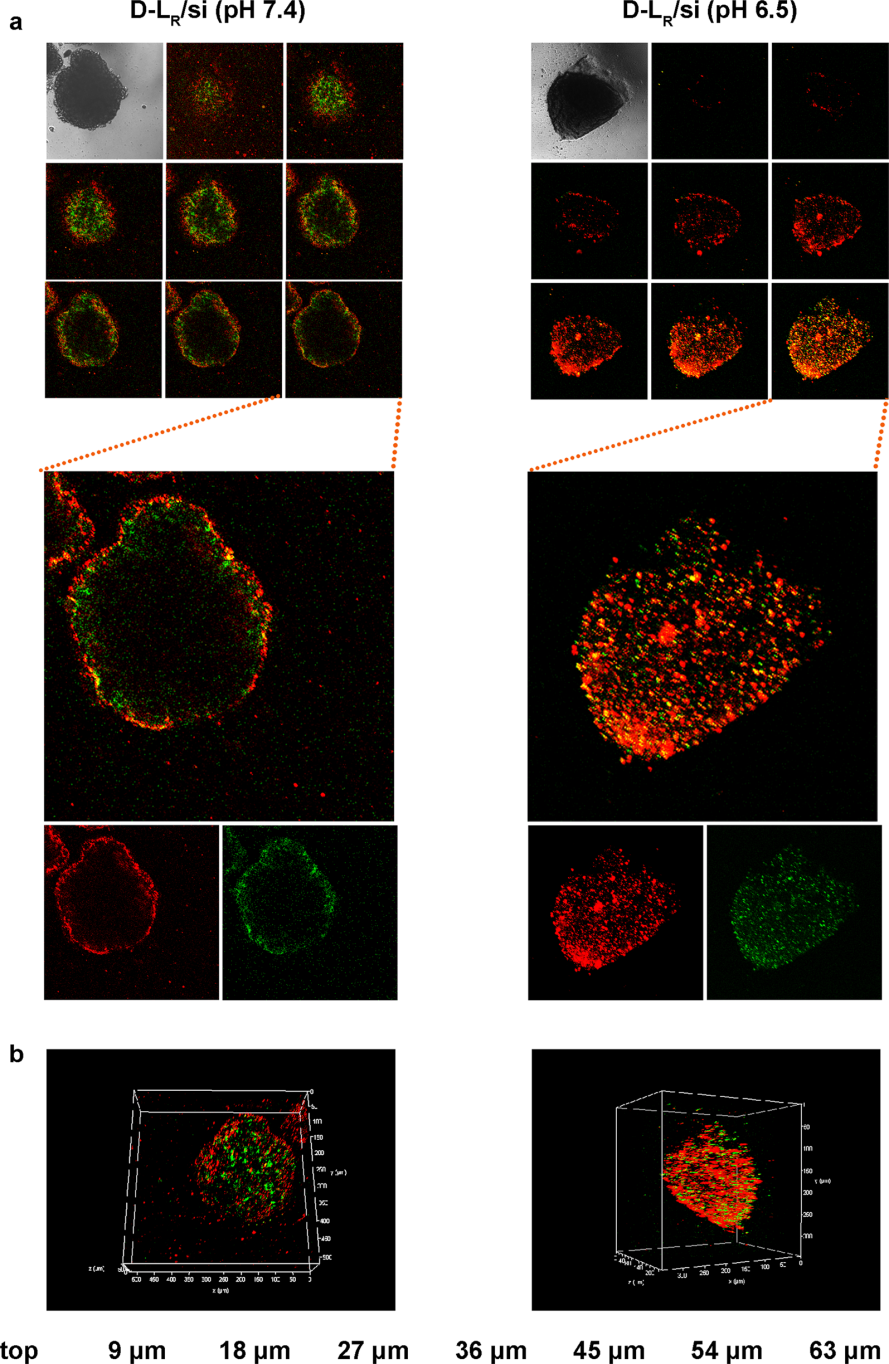
Confocal microscopy images of 3D tumor spheroids of MCF-7 cells after a 24 h incubation with D-L at pH 6.5 and 7.4. **a** Penetration of D-L in MCF-7 tumor spheroids. Z-stack images were obtained from the top to the equatorial plane. Each scanning layer was 8 µm thick, and the total scan depth was 64 µm. **b** 3D reconstruction of all scanned layers. The FAM-siRNA and DiD concentrations were 400 nM and 1.33 μg mL^−1^, respectively. FAM-siRNA fluorescence (green) and DiD fluorescence (red) were recorded

Due to the elevated IFP, drug delivery systems rarely penetrate tumor tissues, which greatly limits their therapeutic application [41]. Three-dimensional tumor spheroids, as approximate models of solid tumors, have been widely applied to bridge the gap between 2D cells and animal models. A three-dimensional tumor spheroid model simulates the pathological environment of solid tumors, such as interstitial pressure, cellular networks and other interior features [42]. Following the construction of MCF-7 tumor spheroids, we assessed the penetration of D-L_R_/si at different pH values (pH 6.5 and 7.4) using CLSM. As exhibited in Fig. 6a, b, a weak fluorescence signal was detected in the core of spheroids after treatment at pH 7.4, indicating that D-L_R_/si did not effectively penetrate the tumor spheroids under this pH condition. In contrast, both FAM-siRNA and DiD exhibit a strong fluorescence signal from the periphery to the interior of the tumor spheroids. CPP decoration is a meaningful method to overcome the obstacle of a high IFP and achieve favorable tumor spheroid penetration [21, 43]. As verified in our previous study [21], the recovered CPP (polyarginine) was significantly conducive to the penetration of nanocarriers to which the peptides are connected. Here, the increased penetration under acidic conditions suggested the activation of DPRP that was incorporated into the surface of D-L_R_/si.

The inhibitory effects on the growth of MCF-7 tumor spheroids were evaluated following the administration of PBS, free siRNA and liposomal formulations. As shown in Additional file 1: Figure S4, the average volume of tumor spheroids treated with PBS (pH 6.5) was twice as high as the initial value following 5 days of culture, indicating rapid growth of the tumor spheroids. In contrast, treatment with free siRNA and D-L_R_/si*N.C.* did not exert a substantial inhibitory effect. Clearly, D-L_R_/si exerted a significantly enhanced antitumor effect (*P* < 0.05) compared with N-L_R_/si. Moreover, once combined with SA-H8-compressed si*PLK-1*, the inhibitory effects of D-L on tumor spheroids were further enhanced, with the tumor spheroid volume decreased to 96% of the initial value after 5 days of treatment. Collectively, the aforementioned results supported the contribution of both DPRP and SA-H8 to inhibiting the growth of tumor spheroids. In addition, the codelivery system D-L_H_/si-DTX exerted a remarkably stronger inhibitory effect on the growth of MCF-7 tumor spheroids (*P* < 0.01). In contrast, the combinatorial administration of D-L_H_/si*N.C.*-DTX and D-L_H_/si only showed moderate inhibition of tumor growth, and no synergistic effect was observed primarily due to the more separate internalization of si*PLK-1* and DTX by tumor cells (Additional file 1: Figure S4). These results revealed the advantages of integrating a combined chemotherapeutic DTX and gene therapeutic si*PLK-1* approach in a single liposomal delivery system.

### Biodistribution of liposomes in tumor-bearing mice

**Fig. 7:**
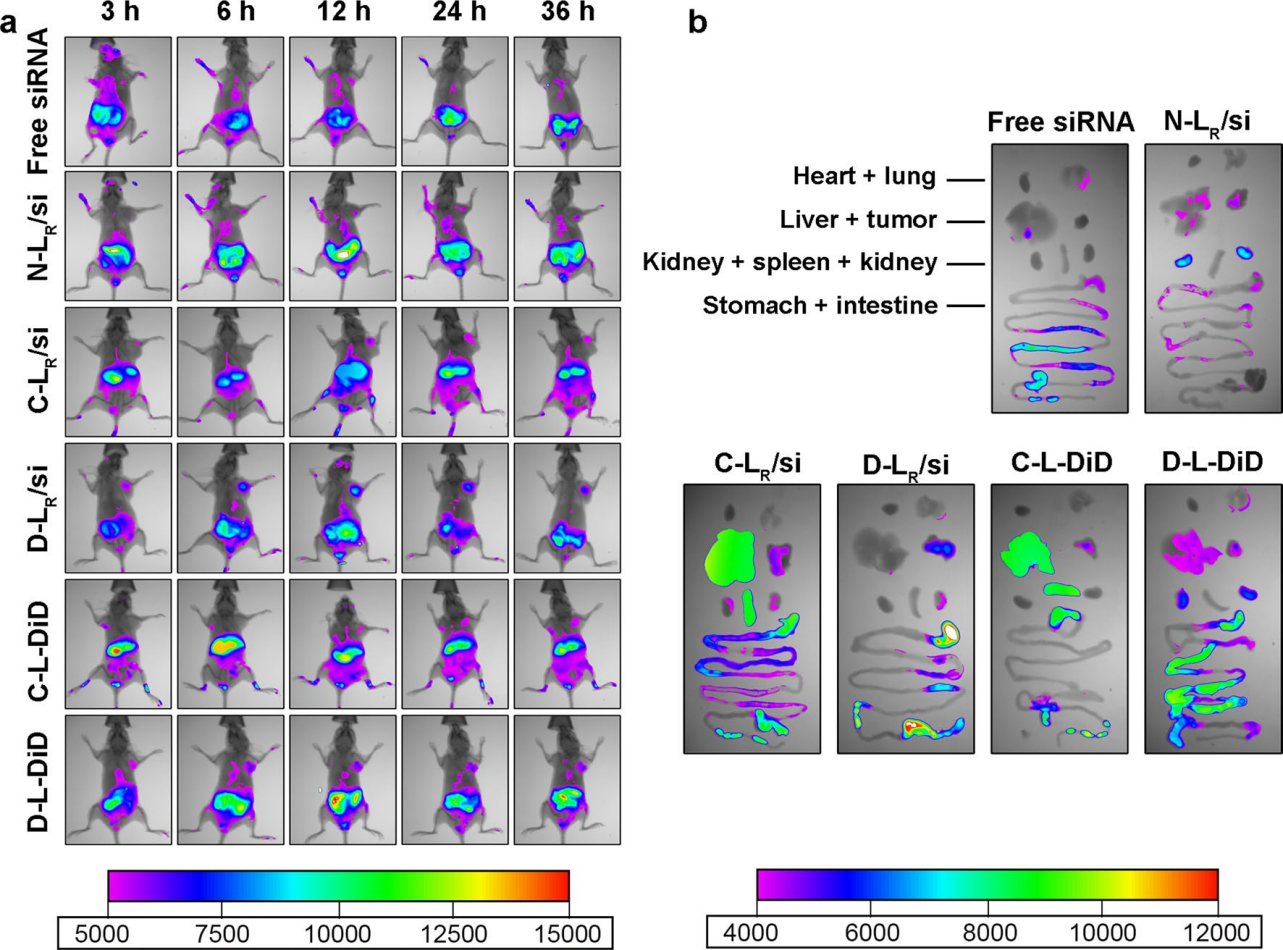
Biodistribution of Cy5-siRNA- or DiD-containing liposomes in mice bearing MCF-7 tumor xenografts. **a** In vivo near infrared fluorescent imaging of MCF-7 tumor-bearing nude mice after administration of Cy5-siRNA- or DiD-loaded formulations. **b** Ex vivo fluorescence images of excised main tissues and organs, including the tumor, heart, liver, spleen, lung, kidney, stomach and intestine, at the end point of observation

In vivo imaging studies were performed to investigate the biodistribution of various siRNA-loaded and DTX-loaded systems in MCF-7 tumor-bearing mice. After the establishment of the MCF-7 xenograft tumor model, samples carrying Cy5-siRNA or DiD were administered individually and the tissue distribution was recorded. Based on whole body imaging (Fig. 7a), tumor accumulation was not detected in mice exposed to free siRNA during the observation period due to enzymatic degradation in the serum during delivery in vivo [44]. After treatment with Cy5-siRNA loaded into N-L_R_/si or C-L_R_/si, fluorescence accumulation was detected to some extent at the tumor site at 6 or 12 h post exposure due to the EPR effect. In contrast, D-L_R_/si significantly increased the accumulation of Cy5-siRNA in the tumor, which was observed even 3 h after the intravenous injection and exhibited high fluorescence intensities during the entire study period. A similar result was obtained after the application of DiD-incorporated samples, which further verified that D-L endowed the payload (DiD) with a noticeably increased tumor selectivity compared to C-L. We excised tumors and major organs at 36 h postadministration for ex vivo imaging to provide clearer evidence for these phenomena (Fig. 7b). The strongest fluorescence was identified in tumors isolated from D-L-treated mice (Fig. 7b), consistent with the results obtained from whole body imaging and confirming the beneficial effect of DPRP on tumor-targeted delivery of both siRNA and DTX. Notably, following the 36-h treatment with C-L, both Cy5-siRNA and DiD were predominantly distributed in the liver and spleen, which was most likely attributed to the strong interaction between oligoarginine and reticuloendothelial system-rich organs, such as the liver and spleen [45–48]. Fluorescence signal was detected in gastrointestinal tissue might be attributed to the fact that mice were not fasted completely during the treatment, and food exhibited fluorescence signal.

### In vivo antitumor efficacy

**Fig. 8:**
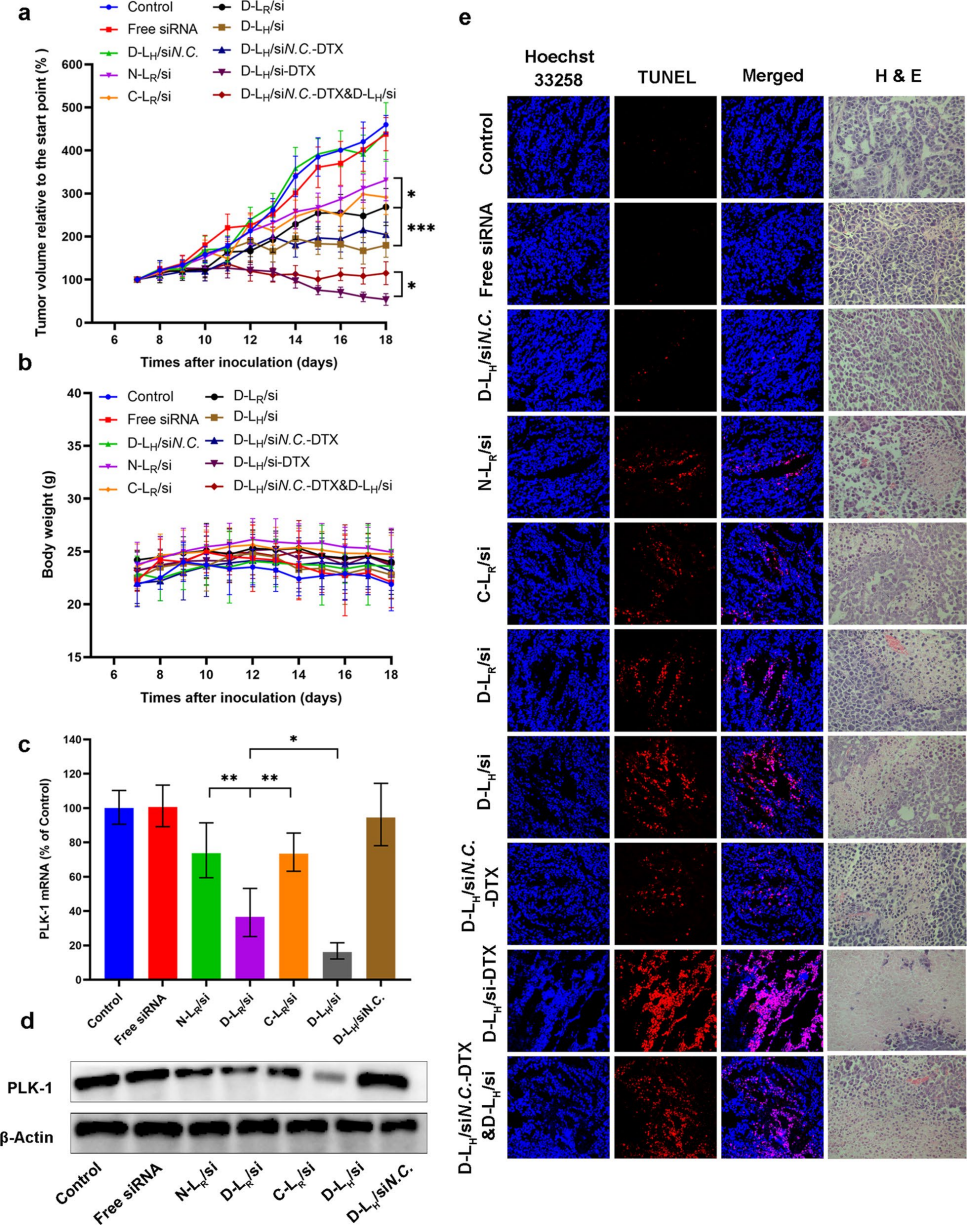
The in vivo antitumor activity of different formulations. **a** The tumor volume and (**b**) body weight changes in MCF-7 tumor-bearing mice after treatments with 5% glucose and various liposomes carrying si*PLK-1* (si*N.C*.) and DTX. Data are presented as the means ± SD (n = 6–7). *P < 0.05 and ***P < 0.001. Expression of the PLK-1 (**c**) mRNA and (**d**) protein in tumors was detected 24 h after the last administration (n = 3). *P < 0.05 and **P < 0.01. **e** TUNEL- and H&E-stained MCF-7 tumor slices from various groups

The substantial accumulation and extended retention of siRNA-formulated D-L in tumors might potentially increase the antitumor efficacy of this therapeutic nucleic acid in vivo. We detected the growth of MCF-7-derived tumors in nude mice following systemic administration to confirm this hypothesis. As illustrated in Fig. 8a, rapid tumor growth was observed in the mice treated with 5% glucose, which led to a > fourfold increase in the mean tumor volume at the end of the test. Treatment with the free siRNA did not show augmented tumor growth inhibition compared to the administration of glucose, likely due to the limited systemic circulation of naked siRNAs and the poor permeability of tumor cells [17]. Moreover, no obvious therapeutic difference was observed between C-L_R_/si and N-L_R_/si, both of which only showed a slight inhibition of tumor growth. In contrast, the retardation of tumor growth was markedly promoted after treatment with D-L_R_/si (*P* < 0.05), revealing that the DPRP modification enhanced the antitumor effects of liposomal si*PLK-1 *in vivo. Furthermore, D-L_H_/si significantly facilitated tumor suppression compared to D-L_R_/si (*P* < 0.001), consistent with the in vitro results described above and indirectly supporting the contribution of SA-H8 to si*PLK-1*-based RNAi. In addition, the administration of D-L_H_ loaded with si*N.C.* failed to inhibit tumor growth compared to the control group, verifying that the antitumor efficacy of liposomal siRNA was achieved in a sequence-specific manner. Collectively, the therapeutic efficacy of the si*PLK-1*-loaded D-L_H_ was significantly superior to that of free or other si*PLK-1* formulations we assessed in vivo, which was positively correlated with the in vitro data obtained from both the analysis of cell apoptosis (Fig. 5c) and evaluation of growth inhibition of tumor spheroids (Additional file 1: Figure S4), supporting the combined processes of DPRP activation in the tumor extracellular microenvironment and the subsequent release of siRNA in the cytoplasm. Compared with other pH-sensitive nanocarriers reported recently [49, 50], the integration of different functional components enables this co-delivery system to respond to pH variations sequentially. Multistage pH-response allows for simultaneous delivery and efficient release of two kinds of drug molecules, which have different molecular size and hydrophilic/lipophilic characteristics.

Many studies and considerable efforts have been devoted to exploring combination therapy; in particular, simultaneous delivery of genes and chemotherapy drugs presents a remarkable potential to enhance antitumor activity [51]. Using MCF-7 xenograft models, we further evaluated the synergistic antitumor efficacy of D-L_H_/si-DTX. Five days after administration, significantly enhanced antitumor activity was observed both in the combinatorial group (D-L_H_/si*N.C.*-DTX & D-L_H_/si) and in the codelivery system D-L_H_/si-DTX compared to all other formulations. Moreover, the application of a codelivery system, D-L_H_/si-DTX, achieved substantially retarded tumor growth at the end of treatment with a relative tumor volume less than 54% (relative to the initial value), which was much smaller than that of the combinatorial group (~ 115%, relative to the initial volume). Based on these results, D-L_H_/si-DTX exerted a synergistic effect through the effective knockdown of PLK-1 and DTX-mediated tumor cell killing. Notably, combinatorial administration did not achieve synchronized pharmacokinetics and biodistribution to exert the combinatorial effects of payloads on tumor growth in vivo [52]. In contrast, D-L_H_/si-DTX unified the pharmacokinetics of coloaded drugs and largely avoided their separate internalization by tumor cells, which is very important for the synergistic inhibitory effects.

Additionally, despite multiple injections of the various formulated siRNAs and/or DTX throughout the treatment period, no apparent change in mouse body weight (Fig. 8b) was observed compared with the control. Hematoxylin–eosin (H&E) staining results revealed that all groups showed excellent biocompatibility and no obvious signs of damage in major organs (Additional file 1: Figure S5). Blood biochemistry analysis results demonstrated that there was no significant difference between liposomes group and the control group (Additional file 1: Figure S6), implying negligible acute or severe toxicity related to the indicated treatment at the tested dose.

### Detection of PLK-1 expression in tumor tissues

We collected tumor tissues to further evaluate the expression of PLK-1 at the mRNA and protein levels using qRT–PCR and western blot analysis, respectively, and to determine whether the inhibition of tumor growth described above was related to PLK-1 gene silencing in tumor cells. As indicated in Fig. 8c and d, either the free siRNA group or D-L_H_/si*N.C.* group exhibited mRNA and PLK-1 protein expression comparable to that of the 5% glucose group. Compared to the C-L_R_/si group, obviously reduced expression of PLK-1 was detected in the D-L_R_/si group at both the mRNA (~ 15% of the control, Fig. 8c) and protein levels (Fig. 8d). Furthermore, the SA-H8-incorporated group, D-L_H_/si, induced stronger PLK-1 gene silencing than the corresponding group including SA-R8 (D-L_R_/si group), along with increased percentages of apoptotic cells (from ~ 47% to ~ 58%). Overall, the results are consistent with the antitumor effect mentioned above, which verified the direct causality between retarded tumor growth and silencing of the PLK-1 gene.

### Pathological evaluation

Cell proliferation and apoptosis in the tumors were investigated by performing H&E staining and terminal deoxynucleotidyl transferase (TdT)-mediated dUTP nick end labeling (TUNEL) assay after treatment (Fig. 8e). H&E-stained sections of tumor tissues from the control, free si*PLK-1* and D-L_H_/si*N.C.* groups exhibited much greater hypercellularity and higher levels of nuclear polymorphisms than all other groups. Among the four therapeutic groups treated with the formulated si*PLK-1*, the tumor tissues from the group treated with D-L_H_/si showed the lowest cell density and the highest level of tumor necrosis. Moreover, D-L_H_/si-DTX treatment resulted in much fewer tumor cells and an elevated level of tumor necrosis in the tumor tissues compared to those of the combinatorial group (D-L_H_/si*N.C.*-DTX & D-L_H_/si). As expected, none of the control treatments (including 5% glucose, free si*PLK-1*, and D-L_H_/si*N.C.*) markedly induced cell apoptosis, as evidenced by the absence of detectable TUNEL-positive tumor cells (red). Simultaneously, the TUNEL assay confirmed that treatment with D-L_H_/si resulted in significantly reduced proliferation and increased apoptosis compared to the other si*PLK-1*-containing liposomal treatments. In contrast to the results obtained from the combinatorial group (D-L_H_/si*N.C.*-DTX & D-L_H_/si), a much larger fraction of apoptotic cells was observed in the D-L_H_/si-DTX group. The TUNEL data were consistent with the trend detected in both the H&E analysis and tumor growth inhibition, which further supported the great potential of both DPRP and SA-H8 for tumor-targeted delivery of siRNA in vivo and verified the synergistic antitumor effect of codelivery treatment.

## Conclusion

In this study, we designed and constructed a novel type of pH-responsive peptide, DPRP. Following the synthesis of DSPE-PEG2000-DPRP, we developed a multistage pH-responsive liposomal platform, D-L/si-DTX, to encapsulate both siRNA and DTX for cancer therapy. In response to both the slightly acidic tumor extracellular microenvironment and the near-neutral cytoplasmic microenvironment, the DPRP-decorated liposomal system exhibited significantly increased cellular uptake, favorable endosome/lysosome escape and effective release of payloads in the cytoplasm. Moreover, the codelivery system achieved augmented PLK-1 silencing, reinforced apoptosis in MCF-7 cells, and increased penetration and inhibition of tumor spheroids. Additionally, systemic administration of D-L/si-DTX displayed markedly improved tumor-selective delivery and significantly increased tumor inhibition activity. Furthermore, the codelivery system D-L/si-DTX exerted excellent synergistic effects on inhibiting cell proliferation, suppressing tumor spheroid growth, and inhibiting tumor growth in vivo, which were superior to those of the combinatorial administration (D-L/si*N.C.*-DTX & D-L/si). In view of the efficient intratumor penetration, cytoplasmic release and synergistic therapeutic effect of two kinds of payloads, lipophilic therapeutic agent (DTX) and nucleic acid-based active biomolecule (siRNA), DPRP-mediated pH-responsive co-delivery platform could be used as an effective tool to synchronize the delivery of various combination of anticancer drugs and therapeutic genes for cancer combination therapy.

## Methods

### Materials

Negatively-charged peptide-4-formylbenzoic acid (NP-FA) and CPP peptides (Ac-CGehGehGehGehG and GrrrrrrrrGC) were ordered at > 95% purity from GL Biochem (Shanghai, China). 1,2-Distearoyl-sn-glycero-3- phosphoethanolamine-N-maleimide (polyethylene glycol) (DSPE-PEG2000-MAL) was purchased from NOF (Tokyo, Japan). Dioleoyl phosphoethanolamine (DOPE) was purchased from NOF (Tokyo, Japan), and cholesterol (Chol) was purchased from Wako (Tokyo, Japan). Cholesteryl hemisuccinate (CHEMS) and SA-R8/SA-H8 were synthesized in our laboratory. DTX was purchased from Nuorui (Beijing, China). Methyl thiazolyl terazolium (MTT) was purchased from TCI (Shanghai, China). The negative control siRNA (si*N.C.*), 6-carboxyfluoresceinaminohexyl (FAM)-labeled negative control siRNA (FAM-siRNA) (antisense strand, 5′-ACGUGACACGUUCGGAGAATT-3′), and siRNA targeting the polo-like kinase 1 (PLK-1) messenger RNA (mRNA) (si*PLK-1*, antisense strand, 5′-GUGAUCUUCUUCAUCAAGGdTdT-3′) were custom-synthesized by GenePharma (Shanghai, China). All primers were synthesized by AuGCT Biotechnology (Beijing, China). Fluorescent probes such as rhodamine-phalloidin, CellLight Early Endosomes-RFP BacMam 2.0, and LysoTracker Red were obtained from Invitrogen/Thermo Fisher Scientific (Waltham, MA, USA). Roswell Park Memorial Institute (RPMI) 1640 medium and fetal bovine serum (FBS) were purchased from Gibco/Thermo Fisher Scientific. Hoechst 33,258 and 4% formaldehyde were supplied by Macgene (Beijing, China). Triton X-100 was purchased from Amresco (Solon, OH, USA).

### Synthesis of DPRP conjugates

The synthesis of the DPRP conjugates is displayed in Fig. 1a. The compound DPRP was synthesized as described by Yang et al. [53], with minor modifications. Briefly, CPP (MW 1571.87, 95.9%) in anhydrous methanol was mixed with NP-FA (MW 1672.63, 93.6%) in the presence of a fivefold molar amount of acetic acid. The reaction mixture was stirred continuously under nitrogen at 30 °C for 12 h. After the product was precipitated in cold anhydrous diethyl ether (DEE)/acetone (8:2, v/v), the resulting solid was collected by centrifugation, rinsed with the same solvent systems, and treated under reduced pressure until dry.

The molecular weight distribution of DPRP (MW 3226.48) was determined using an Applied Biosystems 5600 QTRAP mass spectrometer (ABSciex, Framingham, MA, US) and analyzed in positive ion mode.

### Kinetics of the DPRP hydrolysis

The peptide, final concentration of 2.0 mg mL^−1^, was incubated in phosphate-buffered saline (PBS; 10 mM phosphate, 150 mM NaCl) at pH 6.5 and 7.4 to evaluate the acidic pH-mediated degradation of the imine-containing DPRP. During incubation in buffer solutions at 37 °C, aliquots of the mixture were removed and analyzed using high-performance liquid chromatography (HPLC) at discrete time points (0, 3, 6, 9, 12, 24, and 48 h).

The HPLC analysis was performed using a Kromasil 100–5 C18 column (250 × 4.6 mm, pore size 5 μm) and a Shimadzu LC-20AT HPLC system (Shimadzu, Kyoto, Japan), and the chromatograms were recorded at 220 nm using methanol: water (8:92, v/v) containing 0.01% trifluoroacetic acid (v/v) as a solvent, with a flow rate of 1.0 mL min^−1^ at 30 °C.

### Synthesis of functional conjugates

DSPE-PEG2000-DPRP and DSPE-PEG2000-CPP were synthesized using our published methods [15]. Briefly, the designated peptide (20 mg of thiol-containing DPRP or 10 mg of thiol-containing CPP) was dissolved in 4 mL of methanol. DPRP or CPP solutions were added to a methanol solution of DSPE-PEG2000-MAL and gently stirred at room temperature. After 48 h of stirring under nitrogen protection, the resulting solution was incubated with L-cysteine (10 times the molar ratio of maleimide residues) for 4 h to cap the unreacted maleimide groups. The reaction mixture was dialyzed in a membrane with a molecular weight cutoff of 25,000 (Spectra/Por, Spectrum Labs, Rancho Dominguez, CA) against distilled water (adjusted to pH 10.5–11.0 using 1 M NaOH) for 48 h to remove the excess peptides and quencher. The solution was lyophilized and stored at 20 °C.

The molecular weight distributions of DSPE-PEG2000-DPRP and DSPE-PEG2000-CPP were determined by performing matrix-assisted laser desorption/ionization time-of-flight (MALDI-TOF) mass spectrometry (Bruker Daltonics, Bremen, Germany), using alpha-cyano-hydroxycinnamic acid as the matrix for mass spectrometry analysis.

### Preparation of liposomes

A lipid composition (molar ratio) of DOPE (56%), CHEMS (22%) and Chol (22%) was used to prepare the basic liposomes (SUV). The lipid film was formed in a round bottom flask after evaporation in a rotary evaporator for 60 min at 37 °C. After drying with nitrogen for 1 min to remove the residual solvent, the thin film was hydrated using 10 mL of 10 mM HEPES buffer (HBS) at pH 4.0 (pretreated with DEPC). The lipid dispersion was extruded 5 times through polycarbonate membranes (Whatman, Kent, UK) with a 0.2 μm pore size using an NLI TBX 001 liposome extruder device (Northern Lipids, Burnaby, BC, Canada) to control the size.

The SA-R8/siRNA and SA-H8/siRNA complexes were attained by mixing the siRNA with an SA-R8 (0.1 mg mL^−1^, in HBS) or SA-H8 solution (0.12 mg mL^−1^, in HBS) with vortexing, respectively. Following the incubation of the complexes with SUV for 10 min at room temperature, the system was sonicated for 2 min and cooled in an ice-bath. A NaOH solution (0.1 M) was later added dropwise to the above-mentioned system to adjust the pH value to 7.4.

N-L, C-L and D-L were formed using the postinsertion method. Briefly, a lipid film of DSPE-PEG2000, DSPE-PEG2000/DSPE-PEG2000-CPP or DSPE-PEG2000/DSPE-PEG2000-DPRP was formed in the same manner as described above, and hydrated with HBS (pH 7.8, pretreated with DEPC) to induce the formation of micelles. For the N-L_R_/si, C-L_R_/si or D-L_R_/si preparations, 0.5 mL of micelle solution was added to 3 mL of SA-R8/siRNA loaded SUV at the required molar ratio (3% DSPE-PEG and 5% DSPE-PEG-peptides of total lipids) and incubated for 4 h at 37 °C. D-L_H_/si was prepared using the procedure described above, except that SA-R8 was replaced by SA-H8. In addition, D-L_H_/si-DTX was formulated by incorporating of DTX into the lipid mixture of the SUV film. All of the resulting liposomes were permitted to cool to room temperature before use. Encapsulation efficiency (EE) and drug loading content (DLC) of DTX was determined by HPLC. The HPLC analysis was performed using a Kromasil 100–5 C18 column (250 × 4.6 mm, pore size 5 μm) and a Shimadzu LC-20AT HPLC system (Shimadzu, Kyoto, Japan), and the chromatograms were recorded at 230 nm using methanol: acetonitrile: water (30:50:20, v/v) as a solvent, with a flow rate of 1.0 mL min^−1^ at 30 °C. The EE and DLC values were calculated according to the following equations:

$${\text{EE}}\,\left( \% \right)\, = {\text{A}}_{1} /{\text{A}}_{2} \times 100\%$$

$${\text{DLC}}\,\left( \% \right)\, = {\text{A}}_{1} /{\text{B}} \times 100\%$$

A_1_ is the weight of drug in the liposomes, A_2_ is the weight of drug added, and B is the weight of whole liposomes.

### Characterization of liposomes

The size distribution and zeta-potential of each formulation were determined using DLS (Malvern Zetasizer Nano ZS 90, Malvern, UK). A 1 mL suspension was placed in a DLS cuvette and measured with detection optics arranged at 90°. Three serial measurements were performed for each sample.

The formation of the SA-R8-siRNA complex was evaluated using AGE. Briefly, 2 g of agarose were dissolved in 100 mL of 0.5 × Tris–Borate-EDTA buffer (TBE) with heating and then 10 μL of Exred solution (10,000 ×) were added. The solution was poured into a plate when the temperature was 60 °C. An appropriate amount of 0.5 × TBE was added after 30 min. Subsequently, the comb was gently pulled out vertically and the plate was transferred to an electrophoresis tank. Next, SA-R8 and siRNA (molar ratios: 0, 1, 3, and 5) were mixed with loading buffer and added to the wells. Electrophoresis was performed at 100 V for 30 min. Then the results were visualized and photographed with a gel imager (Bio-Rad).

The morphology of the D-L liposomes was examined using TEM and AFM. For the TEM analysis, 10 μL of the liposomal formulation were uniformly loaded onto a carboncoated copper grid for 1 min, and then the films were negatively stained with 10 μL of a 1% phosphotungstic acid solution for 1 min. Excess sample and stain were absorbed with filter paper and the copper grid was dried for imaging using a HITACHI H-7500 transmission electron microscope (Hitachi, Tokyo, Japan) at 50,000 × magnification. The D-L sample solution was dropped onto the surface of mica and dried under nitrogen for 2 h to acquire AFM topographic images. Particles were observed using a Bioscope Resolve atomic force microscope (Bruker Nano Surfaces, Santa Barbara, CA, USA) in PeakForce quantitative nanomechanical imaging mode. The D-L suspension was imaged at a scan rate of 1 Hz. A Bruker silicon–nitride ScanAsyst Air probe with a spring constant of 0.4 N m^−1^ was used. All images were processed using NanoScope Analysis software (Nanoscope Analysis, Bruker-AXS, Santa Barbara, CA, USA) and Young’s modulus of D-L was calculated by fitting the retract curve using the Derjaguin–Muller–Toropov model.

### Cell culture

The human breast adenocarcinoma cell line (MCF-7 cells) was obtained from the Cell Bank of the Type Culture Collection of the Chinese Academy of Sciences (Shanghai, China) and was grown in RPMI 1640 medium supplemented with 10% FBS, 100 IU mL^−1^ penicillin, and 100 mg mL^−1^ streptomycin. The cells were cultured in a 37 °C humidified incubator with a 5% CO_2_ atmosphere.

Through serial passages, some cells were adapted to grow in the low pH medium (pH 6.5), adjusted with 1 M HCl.

### In vitro cellular uptake

MCF-7 cells grown in pH 6.5 or 7.4 medium were seeded into 6-well plates at a density of 2 × 10^5^ cells per well in 2 mL of complete RPMI 1640 medium and cultured at 37 °C in a 5% CO_2_ humidified atmosphere for 24 h. After the attachment period, the cells were rinsed with PBS and then incubated with pH 6.5 or 7.4 medium containing free siRNA or FAM-siRNA-loaded N-L, C-L and D-L, which were preincubated in serum-free medium at the corresponding pH for 5 h. The ultimate concentration of FAM-siRNA was 100 nM. After treatment for 2 h at 37 °C, the cells were trypsinized and washed with cold PBS containing heparin (500 U mL^−1^). Following two washes with cold PBS, cells were filtered and a flow cytometry analysis was performed with BD FACSCalibur flow cytometer (BD Biosciences, San Jose, CA, USA).

### In vitro confocal imaging

MCF-7 cells (1 × 10^5^ cells) were seeded into a sterile glass-bottomed dish (35 × 10 mm) and incubated in complete RPMI 1640 medium at pH 6.5 and 7.4 for 48 h to assess the cellular uptake by CLSM. After the cells were washed five times with PBS, serum-free medium containing free or liposomal FAM-siRNA was introduced as described in the earlier paragraph (N-L, C-L, and D-L (D-L_R_/FAM-si and D-L_H_/FAM-si)). The final concentration of FAM-siRNA in the culture medium was 200 nM. The cells were then incubated at 37 °C for 3.5 h and washed four times with cold PBS containing heparin (500 U mL^−1^). Cells were fixed with 4% formaldehyde for 10 min, followed by three 5-min rinses with cold PBS. Cells were stained with rhodamine-phalloidin for 20 min after permeabilization with 0.1% Triton X-100 in PBS to image F-actin. Finally, the nuclei were labeled with Hoechst 33,258 at 37 °C for another 20 min and imaged using a Leica TCS SP8 confocal platform (Leica Microsystems Inc., Mannheim, Germany). FAM-siRNA, rhodamine-phalloidin, and Hoechst 33,258 were excited with 494, 540, and 352 nm lasers, respectively.

The internalization and endosomal release of the liposomal FAM-siRNA were characterized by performing CLSM. MCF-7 cells were seeded in a sterile glass-bottomed dish (35 × 10 mm) at a density of 6 × 10^4^ cells per well and cultured in complete RPMI 1640 medium at pH 6.5. MCF-7 cells were preincubated with CellLight Early Endosomes-RFP BacMam 2.0 (20 particles per cell, as recommended by the supplier) for 24 h at 37 °C to stain the early endosomes. Late endosomes/lysosomes were labeled with 500 nM LysoTracker Red (Invitrogen/Molecular Probes, CA, USA) for 0.5 h. The following day, the cells were incubated with acid-pretreated D-L (SA-R8/FAM-siRNA or SA-H8/FAM-siRNA) for 3 or 6 h at pH 6.5. Subsequently, the cells were rinsed three times with cold PBS containing heparin (500 U mL^−1^) and then fixed and subjected to nuclear staining.

MCF-7 cells were incubated with double-labeled liposomes (containing 400 nM FAM-siRNA and 680 ng mL^−1^ DiD) that had been pretreated as described above at 37 °C for 3 h and 6 h to observe the time-dependent changes in the intracellular uptake and distribution of FAM-siRNA or liposomes, respectively. Images of fluorescent cells were also recorded.

Confocal microscopy images were acquired using a Leica confocal platform with a 63 × oil immersion objective at an excitation wavelength of 494 nm for FAM-siRNA, 555 nm for CellLight Early Endosomes-RFP BacMam 2.0, 561 nm for LysoTracker Red, 620 nm for DiD dye, and 352 nm for Hoechst 33,258.

### In vitro transfection and analysis of gene silencing

MCF-7 cells were seeded into 25 cm^2^ tissue culture flasks at a density of 1.5 × 10^6^ cells/flask in 4 mL of complete RPMI 1640 medium. After a 24 h incubation at 37 °C in a humidified atmosphere with 5% CO_2_, the medium was replaced with fresh serum-free medium containing free siRNA or siRNA-loaded samples (N-L, C-L, or D-L (D-L_R_/si and D-L_H_/si)). The final concentration of the siRNA (si*PLK-1* or si*N.C.*) utilized in the experiment was 100 nM. Following a 5 h incubation, the medium was replaced with complete medium and cells were cultured for an additional 48 h (for mRNA assay) or 72 h (for protein quantification) at 37 °C. Subsequently, the PLK-1 mRNA and protein levels were determined using quantitative reverse transcription polymerase chain reaction (qRT–PCR) and western blot analysis, respectively.

For qRT–PCR assessment, cells were collected and total RNA was extracted from transfected cells using TRIzol reagent (Tiangen, China) according to the manufacturer’s protocol. First strand cDNAs were synthesized from 2 μg of total RNA with a Quantscript RT Kit (first strand cDNA synthesis kit) (Tiangen, China). After cDNA synthesis, 4 μL of cDNA templated were subjected to qRT–PCR analysis of PLK-1 and glyceraldehyde 3-phosphate dehydrogenase (GAPDH) expression using the SuperReal Premix SYBR Green kit (Tiangen, China). Following analysis using the CFX 96 Touch Real-Time PCR Detection System (Bio-Rad), the relative gene expression was quantified using the 2^−∆∆Ct^ method. Data are presented as the fold change in PLK-1 expression normalized to the housekeeping gene GAPDH as the endogenous reference and relative to the untreated control cells. The primers used for PCR amplification were as follows: GAPDH forward: 5ʹ-GGGTGTGAACCATGAGAAGT-3ʹ; GAPDH reverse: 5ʹ-GACTGTGGTCATGAGTCCT-3ʹ; PLK-1 forward: 5ʹ-CGAGGTGCTGAGCAAGAAAGGGC-3ʹ; and PLK-1 reverse: 5ʹ-CCACGGGGTTGATGTGCTTGGGA-3ʹ. The cycling procedure was as follows: 95 °C for 15 min, followed by 40 cycles of 95 °C for 10 s and 61 °C for 30 s. The specificity was verified by performing a melting curve analysis and agarose gel electrophoresis.

For the western blot assay, the transfected cells were first washed with ice-cold PBS three times and then lysed in radioimmunoprecipitation assay buffer (Bestbio. Co. Ltd., Shanghai, China) containing phenylmethanesulfonylfluoride (PMSF). The resulting cell suspension was incubated on ice for 30 min with vortexing every 5 min. The lysates were collected by centrifugation at 14,000 rpm for 10 min at 4 °C. Subsequently, the protein concentration was determined using the bicinchoninic acid protein assay (MultiSciences Biotech, Beijing, China). After separation by 10% sodium dodecyl sulfate–polyacrylamide gel electrophoresis, the total protein (50 mg) was transferred (at 250 mA for 2.5 h) to Immobilon-P membranes (Millipore, Bedford, MA, USA). Membranes were blocked with 5% bovine serum albumin (BSA) in Tris-buffered saline with Tween-20 (TBST) for 1 h at room temperature and incubated overnight at 4 °C with an anti-PLK-1 monoclonal antibody (Cell Signaling Technology Inc., Danvers, MA, USA; 1:1,000) or rabbit anti-beta-actin antibody (Antibody Revolution Inc., San Diego, CA, USA; 1:2,000) as the internal control in TBST containing 5% BSA. Next, the membrane was incubated with a horseradish peroxidase-linked anti-rabbit IgG antibody (Cell Signaling Technology Inc.; 1:3,000) in 5% BSA for 1 h at ambient temperature, followed by imaging using the Molecular Imager ChemiDoc XRS + system (Bio-Rad).

### Cell apoptosis assay

MCF-7 cells growing in pH 6.5 or 7.4 medium were cultured in 25 cm^2^ tissue culture flasks at a density of 6 × 10^5^ cells per flask in 4 mL of complete RPMI 1640 medium, as mentioned above. After 24 h of culture at 37 °C in a 5% CO_2_ humidified atmosphere, the cells were washed with PBS and exposed to fresh serum-free medium containing free si*PLK-1* or si*PLK-1* (or si*N.C.*, only for D-L)-loaded N-L, C-L and D-L. The final concentration of siRNA used in the experiment was 100 nM. Following a 6 h incubation, the medium was replaced with complete medium for a routine culture of 72 h at 37 °C. Subsequently, the cells were collected and stained with the Annexin V-FITC apoptosis detection kit (KeyGEN, Nanjing, China) according to the manufacturer’s instructions and were immediately analyzed using a BD FACSCalibur flow cytometer by collecting 10,000 events (excitation 488 nm; emission 530 nm).

Cells were incubated with D-L_H_/si-DTX for 48 h and then processed using the same procedures described above to assess the effect of the codelivery of DTX and the siRNA on apoptosis. The final concentrations of si*PLK-1* and DTX were 100 nM and 0.1 µg mL^−1^, respectively.

### Cell proliferation assay

MCF-7 cells were cultured with the samples over a wide range of concentrations to evaluate the cytotoxicity of different D-L samples. Cell viability was measured using the MTT assay. Cells (5 × 10^3^ cells/well) were seeded into 96-well plates and incubated at 37 °C and 5% CO_2_ before the experiment, and a total volume of 200 μL was used. The old medium was replaced with medium containing D-L (100 nM siRNA and 0.01 µg mL^−1^ DTX) or the controls after 12 h of incubation. Untreated cells cultured in growth medium were used as the blank control. After 72 h, 20 μL of MTT solution (5 mg mL^−1^) were added to each well and incubated with cells for another 4 h. Next, the solution was removed, and 100 μL of DMSO were added to dissolve the MTT formazan crystals. The absorbance was measured at a wavelength of 490 nm using a microplate reader (Synergy 4, Bio Tec, USA). Cell viability (%) was defined as the percentage of the absorbance of the wells containing the cells incubated with the sample suspension to the blank control. All data are presented as the means of four measurements (± SD). The experiment was repeated three times.

### Penetration and inhibition of three-dimensional tumor spheroids

Three-dimensional tumor spheroids of MCF-7 cells were prepared using hanging drop method, as previous described [54]. Briefly, 200 μL of agarose gel solution (2%, w/v) were heated at 80 °C and added to a 48-well plate. After cooling and solidifying, 500 μL of complete RPMI 1640 medium (pH 6.5 or 7.4) were added to each well. Twenty microliters of a cell suspension (1 × 10^3^ cells) were suspended on the lid of a 48-well culture plate to induce sufficient cell aggregation. The cells were incubated in a 37 °C humidified incubator with a 5% CO_2_ atmosphere followed by 72 h of culture. The resulting cellular aggregates were transferred to the corresponding wells and grown for another 48 h.

MCF-7 tumor spheroids (300 μm in diameter) were incubated for 24 h with D-L (400 nM FAM-siRNA and 1.33 μg mL^−1^ DiD) at pH 6.5 and 7.4 to evaluate the penetration ability. After two washes with cold PBS, the tumor spheroids were transferred to a chambered coverslip and examined using CLSM. The tumor spheroids were scanned from the top to the equatorial plane to acquire Z-stack images. Each scanning layer was 8 µm thick, and the total scan depth was 64 µm.

MCF-7 tumor spheroids were incubated with PBS, free si*PLK-1* and si*PLK-1-* or si*N.C.*-loaded N-L, C-L and D-L (200 nM siRNA and 2 μg mL^−1^ DTX) for 5 days to evaluate the inhibitory effect on proliferation. The spheroid volume was monitored with a 10 × objective lens using an inverted phase microscope (Motic, Xiamen, China). The major (d_max_) and minor (d_min_) diameters of each tumor spheroid were recorded, and the volume was calculated with the formula: V = 0.5 × d_max_ × d_min_^2^. The percent change in the tumor spheroid volume ratio was calculated using the following equation: R = (V_i_/V_0_) × 100%, where V_i_ is the tumor spheroid volume after treatment and V_0_ is the tumor spheroid volume before treatment.

### Animals

Female BALB/c nude mice (6 weeks old) were purchased from the Vital River Laboratory Animal Center (Beijing, China) and fed a rodent diet. All procedures involving animal housing and treatment were approved by the Institutional Authority for Laboratory Animal Care of Hebei Medical University.

### In vivo imaging

MCF-7 cells were subcutaneously injected into BALB/c mice to establish a subcutaneous xenograft breast tumor-bearing mouse model. When the tumor volume reached approximately 200 mm^3^, the mice were intravenously injected with 200 μL of free Cy5-siRNA or different liposomes (N-L, C-L, or D-L) containing Cy5-siRNA at 0.28 mg kg^−1^ and formulated DiD (C-L and D-L) at 0.1 mg kg^−1^. Subsequently, the acquisition of images of fluorescence signals from the whole body was performed using a Kodak in vivo imaging system (Kodak In Vivo Imaging System FX Pro, Carestream Health, USA) at the indicated time points (3, 6, 12, 24 and 36 h after injection). At the end point, the mice were sacrificed by cervical dislocation, and the major organs and tissues, including the heart, lung, liver, spleen, kidney, stomach, intestine and tumor, were collected and examined as described above.

### Tumor suppression study

A xenograft tumor model was built by subcutaneously injecting MCF-7 cells, as described above, to assess the antitumor efficacy. After the tumors had grown to approximately 100 mm^3^, the mice were randomly divided into 10 groups (n = 6–7) and treated with 5% glucose (Control), free si*PLK-1*, various liposomal formulations (N-L, C-L, and D-L) carrying siRNA or/and DTX by intravenous injection once every other day for 10 d. The doses of siRNA and DTX in each injection were 0.28 mg kg^−1^ and 0.86 mg kg^−1^, respectively. The body weight and tumor size were measured at least once every 2 days throughout the postexposure period. The major (D_max_) and minor (D_min_) diameters of each tumor were recorded, and the volume was calculated with the formula: V_T_ = 0.5 × D_max_ × D_min_^2^. Relative tumor volume (RTV) was calculated using the formula RTV = (V_a_/V_0_) × 100%, where V_a_ is daily tumor volume, V_0_ is initial tumor volume.

### Detection of PLK-1 expression in tumor tissues

Tumor tissues were removed 24 h after the last administration to analyze PLK-1 expression in vivo. Tumor fragments (100 mg) were processed for total mRNA or protein extraction followed by qRT–PCR and western blot assays, respectively. The extracted mRNA samples were standardized to the same absorbance value of 260 nm and the expression of the PLK-1 mRNA was detected using qRT–PCR as described above. The selected tumor tissues were homogenized in 1 mL of RIPA lysis buffer (20 mM Tris–HCl (pH 7.4), 150 mM NaCl, 1% Triton X-100, 10 mM KCl, 1.5 mM MgCl_2_, 1 mM DTT, 100 mM AEBSF, 100 μM aprotinin, 5 mM bestatin, 1.5 mM E64, 2 mM leupeptin, 1.5 mM pepstatin A, cyclosporin A, sodium fluoride, beta-glycerophosphoric acid disodium salt, sodium orthovanadate, disodium molybdate dihydrate, and sodium pyrophosphate) (BestBio, China) supplemented with PMSF (1 mM) to evaluate PLK-1 protein levels. The lysates were incubated on ice for a total of 30 min and vortexed every 5 min. After purification and quantification, the protein levels were determined using western blot analysis as described above.

### Pathological evaluation

For the histological analysis of tumor tissues and organs, the mice were sacrificed 24 h after the last administration, and both the tumor tissues and the major organs, including heart, lung, liver, spleen and kidney, were excised from one mouse randomly selected from each group. After 4% formaldehyde fixation, the sectioned specimens underwent H&E staining and histological evaluation using an optical microscope.

TUNEL staining for apoptosis was conducted on the specimens using the TRITC staining in situ Apoptosis Detection Kit (KeyGEN, Nanjing, China) according to the manufacturer’s protocol. Briefly, frozen sections were fixed with 4% formaldehyde for 30 min at room temperature and washed three times with PBS. Following an incubation in permeabilization solution (freshly prepared 1% Triton X-100 in PBS, pH 7.4) for 5 min, the sections were rinsed three times with PBS followed by treatment with 3% hydrogen peroxide (diluted in methanol) for 10 min at ambient temperature. Each sample was labeled with 50 μL of reaction mixture (45 μL of equilibration buffer, 1.0 μL of TRITC-5-dUTP and 4.0 μL of the TdT enzyme) at 37 °C in a dark and humidified atmosphere. For a positive control, 100 μL of DNase I reaction solution were added to the sample and treated for 30 min at 37 °C before the introduction of the TdT enzyme. For the negative control, the TdT enzyme was excluded from the labeling reaction mixture. Nuclei were stained with Hoechst 33,258 for 25 min at 37 °C, and the sections were examined under a confocal laser scanning microscope (Leica, Heidelberg, Germany).

### Statistical analysis

All data are presented as the means ± standard deviations (SD) from at least three repeated experiments. Differences between any two groups were determined using ANOVA. *P* < 0.05 was considered statistically significant.

## Supplementary Information

**Additional file 1: Methods.** Blood analysis. **Results and discussion.** Cell proliferation assay. **Figure S1.** pH-sensitive profiles of DPRP. The responses of DPRP were plotted against the incubation time at 37 °C. Data are presented as the means ± SD (n = 3). *P < 0.05. **Figure S2.** Characterization of SA-R8 and SA-H8. a Principle of SA-R8/SA-H8 synthesis. b The positive ion electrospray ionization mass spectrum of SA-R8. c MALDI-TOF mass spectrum of SA-H8. **Figure S3.** Cytotoxicity analysis of different formulations. Data are presented as the means ± SD (n = 3). **P < 0.01. **Figure S4.** The inhibitory effect of different formulations on the growth of MCF-7 tumor spheroids. Data are presented as the means ± SD (n = 6). *P < 0.05 and **P < 0.01. **Figure S5.** H&E staining of various organ tissues from MCF-7 tumor-bearing mice after treatment with different formulations. **Figure S6.** Blood biochemistry analysis of mice treated with 5% Glucose, free siRNA, D-L_H_/si-DTX and D-L_H_/si. ALT: Alanine aminotransferase; AST: Aspartate aminotransferase; CREA: Creatinine; CK: Creatine Kinase. Data are presented as the means ± SD (n = 3).

## Data Availability

All data generated or analyzed during this study are included in this manuscript.

## Abbreviations

AFM: Atomic force microscopy
AGE: Agarose gel electrophoresis
BSA: Bovine serum albumin
CHEMS: Cholesteryl hemisuccinate
CHOL: Cholesterol
CLSM: Confocal laser-scanning microscopy
CPPs: Cell-penetrating peptides
DEE: Diethyl ether
DOPE: Dioleoyl phosphoethanolamine
DPRP: Dual pH-responsive peptide
DTX: Docetaxel
EPR: Enhanced permeability and retention
FAM: 6-Carboxyfluoresceinaminohexyl
FBS: Fetal bovine serum
GAPDH: Glyceraldehyde 3-phosphate dehydrogenase
HBS: HEPES buffer
H&E: Hematoxylin–eosin
HIV: Human immunodeficiency virus
HPLC: High-performance liquid chromatography
IFP: Interstitial fluid pressure
MTT: Methyl thiazolyl terazolium
NP-FA: Negatively-charged peptide-4-formylbenzoic acid
PBS: Phosphate-buffered saline
PDI: Polydispersity index
PLK-1: Polo-like kinase-1
PMSF: Phenylmethanesulfonylfluoride
RNAi: RNA interference
RPMI: Roswell Park Memorial Institute
SD: Standard deviations
si*N.C.*: Negative control siRNA
si*PLK-1*: Polo-like kinase-1 specific siRNA
siRNA: Small interfering RNA
TAT: Transactivator of transcription
TBE: Tris–borate-EDTA buffer
TBST: Tris-buffered saline with Tween-20
TEM: Transmission electron microscopy
TUNEL: Terminal deoxynucleotidyl transferase-mediated dUTP nick end labeling
