# Supplementary material for: Multistage pH-responsive codelivery liposomal platform for synergistic cancer therapy
Source: J Nanobiotechnology. 2022 Apr 2;20:177. doi: 10.1186/s12951-022-01383-z (PMC8976966; doi:10.1186/s12951-022-01383-z)
Supplement: Supplementary file 1 — Additional file 1: Methods. Blood analysis. Results and discussion. Cell proliferation assay. Figure S1. pH-sensitive profiles of DPRP. The responses of DPRP were plotted against the incubation time at 37 °C. Data are presented as the means ± SD (n = 3). *P < 0.05. Figure S2. Characterization of SA-R8 and SA-H8. a Principle of SA-R8/SA-H8 synthesis. b The positive ion electrospray ionization mass spectrum of SA-R8. c MALDI-TOF mass spectrum of SA-H8. Figure S3. Cytotoxicity analysis of different formulations. Data are presented as the means ± SD (n = 3). **P < 0.01. Figure S4. The inhibitory effect of different formulations on the growth of MCF-7 tumor spheroids. Data are presented as the means ± SD (n = 6). *P < 0.05 and **P < 0.01. Figure S5. H&E staining of various organ tissues from MCF-7 tumor-bearing mice after treatment with different formulations. Figure S6. Blood biochemistry analysis of mice treated with 5% Glucose, free siRNA, D-LH/si-DTX and D-LH/si. ALT: Alanine aminotransferase; AST: Aspartate aminotransferase; CREA: Creatinine; CK: Creatine Kinase. Data are presented as the means ± SD (n = 3). [file 12951_2022_1383_MOESM1_ESM.docx]

Additional file 1 Information

**Multistage pH-Responsive Codelivery Liposomal Platform for Synergistic Cancer Therapy**

*Ting Zhao, Ce Liang, Yanrong Zhao, Xiangdong Xue, Zhao Ma, Jinlong Qi, Haitao Shen, Shaokun Yang, Jia Zhang, Qingzhong Jia, Qing Du, Bai Xiang*, Deying Cao*, Hailin Zhang*, Xianrong Qi*

**Methods**

**Blood analysis**

Healthy female BALB/c mice were randomly divided into five groups (n = 3) and given an intravenous injection of 5% Glucose, free si*PLK-1*, D-L_H_/si-DTX and D-L_H_/si. The doses of siRNA and DTX in each injection were 0.28 mg kg^-1^ and 0.86 mg kg^-1^, respectively. After three injections, the blood was collected 24 h post the final injection and serum isolated for blood biochemical analysis.

**Results and discussion**

**Cell proliferation assay**

The proliferation of MCF-7 cells was determined using the methyl thiazolyl terazolium (MTT) assay. As shown in Figure S3, treatment with the codelivery system D-L_R_/si-DTX exerted a greater inhibitory effect than that of D-L_R_/si and D-L_R_/DTX. Moreover, cell viability was significantly reduced when MCF-7 cells were treated with D-L_R_/si-DTX compared to the combinatorial treatment (D-L_R_/si*N.C.*-DTX & D-L_R_/si) (*P* < 0.01), which correlated well with the result of apoptosis analysis (Fig. 4d) and revealed a synergistic effect on cancer cells due to simultaneous delivery of si*PLK-1* and DTX.

Figures


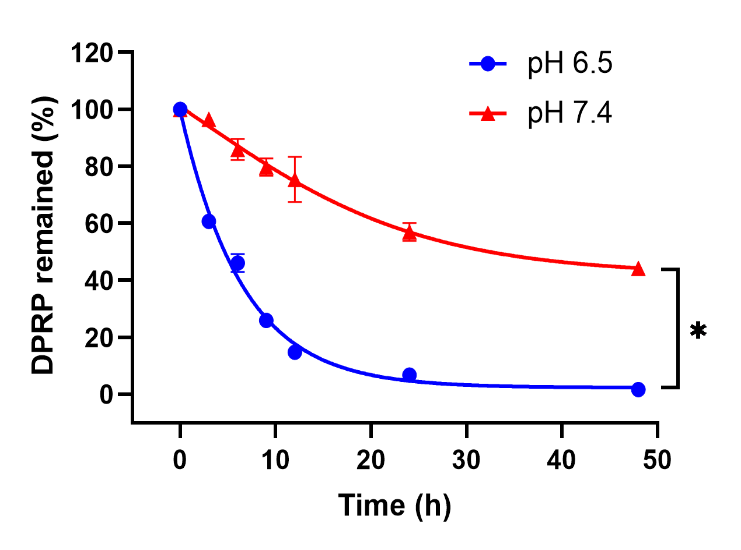


**Figure S1** pH-sensitive profiles of DPRP. The responses of DPRP were plotted against the incubation time at 37 °C. Data are presented as the means ± SD (n = 3). **P* < 0.05


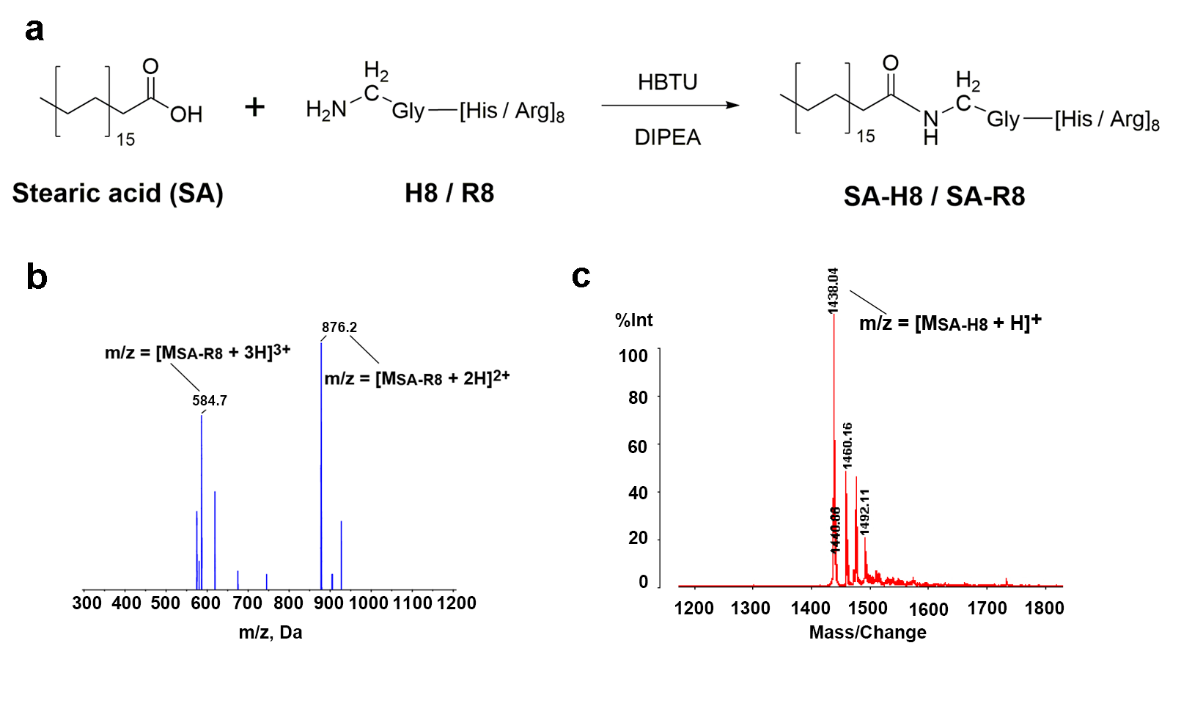


**Figure S2** Characterization of SA-R8 and SA-H8. **a** Principle of SA-R8/SA-H8 synthesis. **b** The positive ion electrospray ionization mass spectrum of SA-R8. **c** MALDI-TOF mass spectrum of SA-H8.


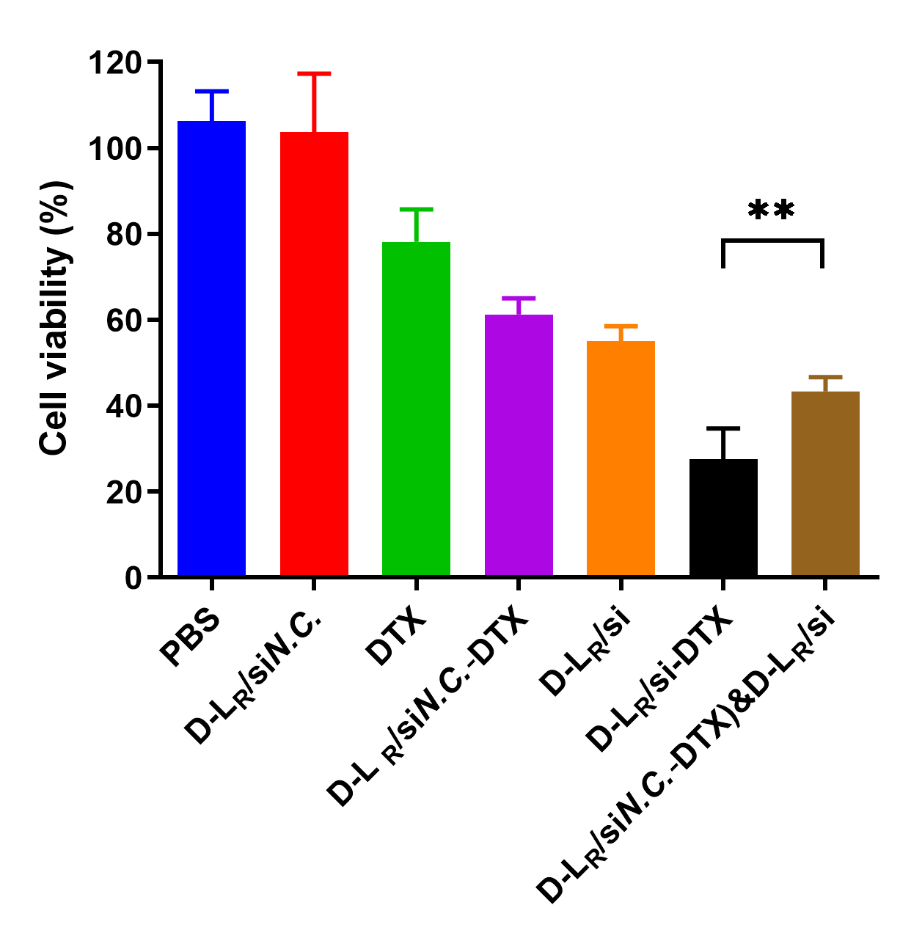


**Figure S3** Cytotoxicity analysis of different formulations (n = 3). ***P* < 0.01.


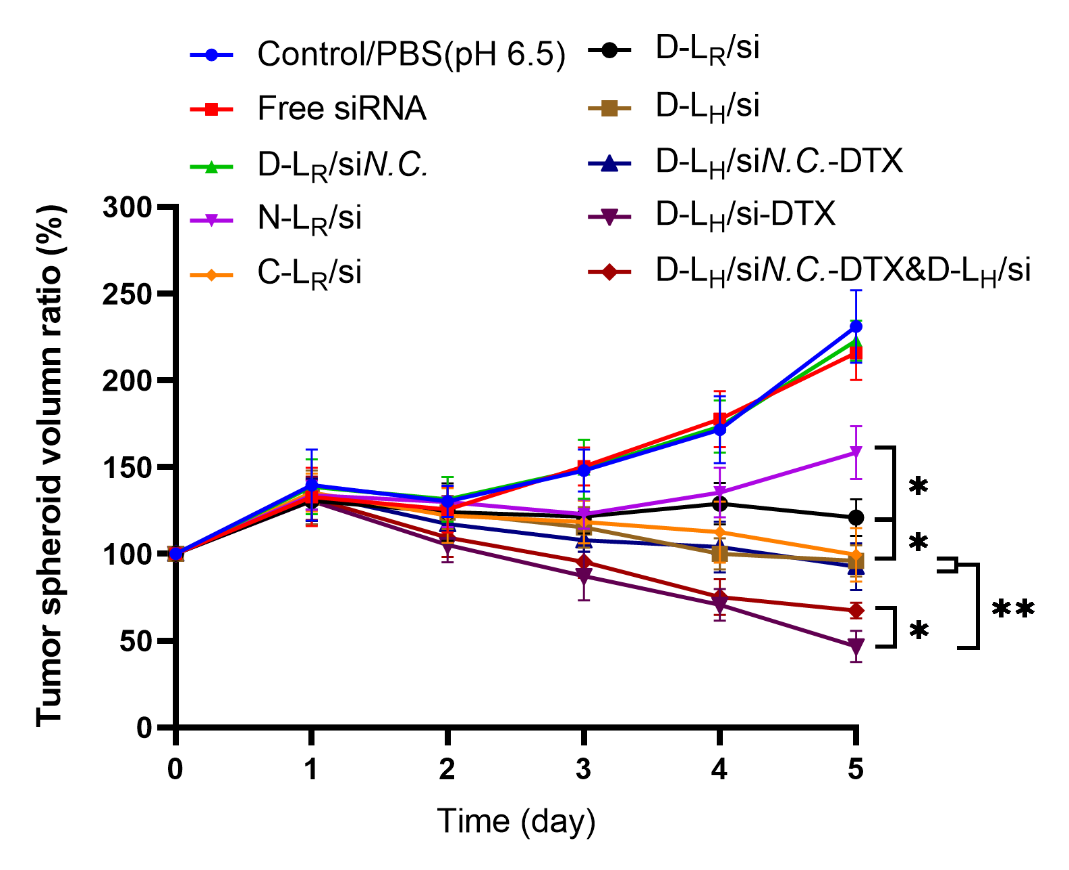


**Figure S4** The inhibitory effect of different formulations on the growth of MCF-7 tumor spheroids. Data are presented as the means ± SD (n = 6). **P* < 0.05 and ***P* < 0.01


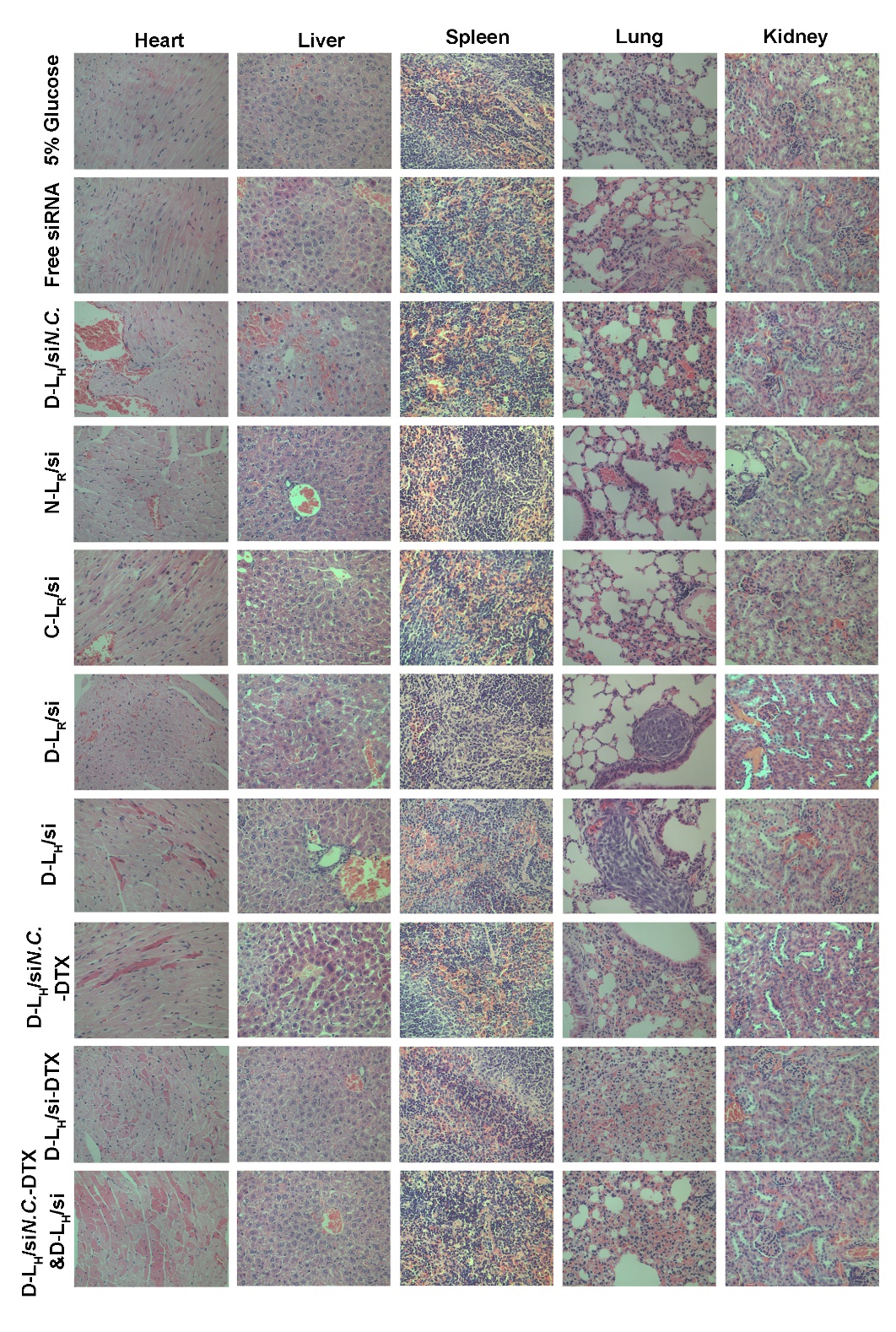


**Figure S5** H&E staining of various organ tissues from MCF-7 tumor-bearing mice after treatment with different formulations.


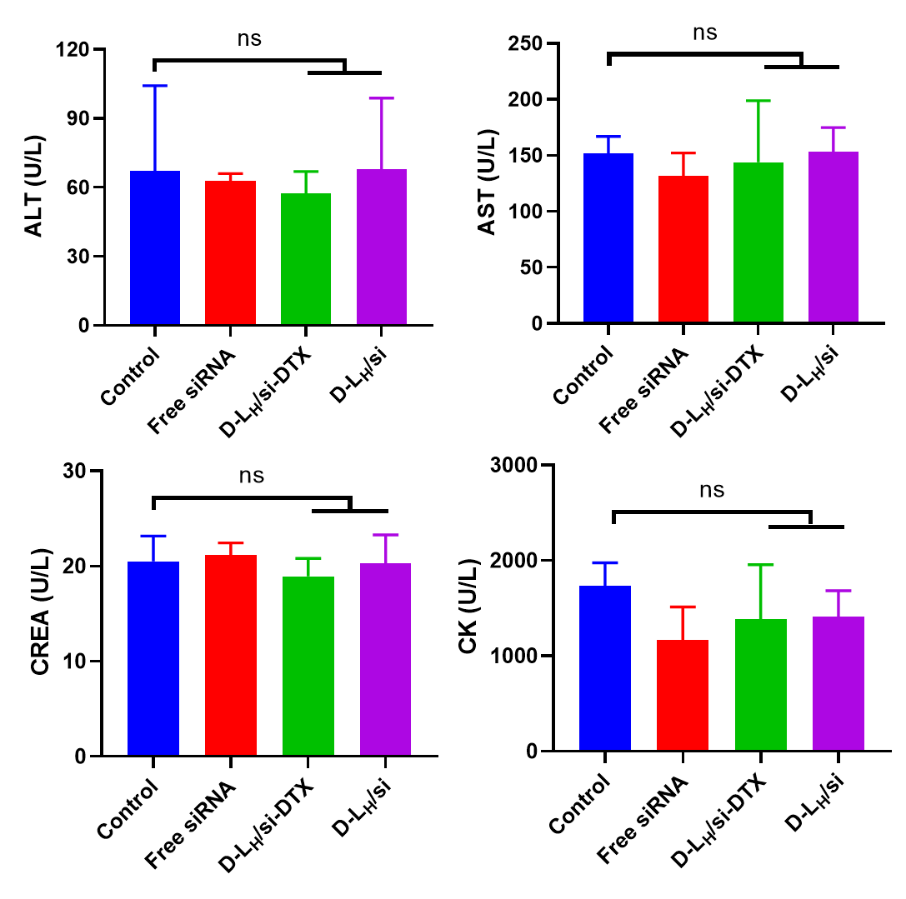


**Figure S6** Blood biochemistry analysis of mice treated with 5% Glucose, free siRNA, D-L_H_/si-DTX and D-L_H_/si. ALT: Alanine aminotransferase; AST: Aspartate aminotransferase; CREA: Creatinine; CK: Creatine Kinase. Data are presented as the means ± SD (n=3).
